# Quantified Activity Patterns for Young Children in Beach Environments Relevant for Exposure to Contaminants

**DOI:** 10.3390/ijerph18063274

**Published:** 2021-03-22

**Authors:** Alesia Ferguson, Ashok Dwivedi, Foluke Adelabu, Esther Ehindero, Mehdi Lamssali, Emmanuel Obeng-Gyasi, Kristina Mena, Helena Solo-Gabriele

**Affiliations:** 1 Department of Built Environment, North Carolina Agricultural and Technical State University, Greensboro, NC 27411, USA; dwivedi.ashok@gmail.com (A.D.); fradelabu@aggies.ncat.edu (F.A.); eehindero@aggies.nat.edu (E.E.); mlamssali@aggies.ncat.edu (M.L.); eobenggyasi@ncat.edu (E.O.-G.); 2 Department of Epidemiology, Human Genetics and Evironmental Sciences, University of Texas Houston School of Public Health, Houston, TX 77030, USA; Kristina.D.Mena@uth.tmc.edu; 3 Department of Civil Architectural and Environmental Engineering, University of Miami, Coral Gables, FL 33124, USA; hmsolo@miami.edu

**Keywords:** videotaping, videotranslation, children’s exposure activities, micro-level time series, micro activity patterns

## Abstract

In a study to evaluate beach play activities, 120 children were videotaped to observe and quantify factors that could influence their exposure to contaminants in the beach environment. Children aged 1 to 6 years were followed by researchers with video cameras at beaches (two in Miami, Florida and two in Galveston, Texas) for approximately one hour each. Factors evaluated included time spent in various beach locations, various activities engaged in, and various surfaces contacted (including contacts by hand and mouth). Activities recorded in the videos were transcribed to text files to allow for quantitative analyses. Across all sexes, age groups, and beaches, Wading was the most common activity and Seawater was the most common location where children played. The left hand was found to not be in contact with objects most of the time, while the right hand, considered the most dominant hand in most cases, contacted Plastic-Toys the most. Although activity patterns collection through videotaping and videotranslation can be labor-intensive, once collected, they can be widely useful for estimates of exposures to all contaminants in the beach environment (e.g., microorganisms and chemicals) as well as UV exposure, with considerations for whether the contaminants are found in water, sand or both. These activity patterns were collected to potentially look at exposures following the Deepwater Horizon 2010 Spill.

## 1. Introduction and Background

Contaminants (e.g., microorganisms, oil spill contaminants) can be found at beach areas where children play [1]. Children are more vulnerable to adverse toxicological outcomes because they are potentially more exposed to contaminants due to their unique activity patterns that increase contact (i.e., rolling in sand), have higher uptake rates (i.e., breathing rates), and are at critical stages of childhood for developing organs [2]. The magnitude of children exposures to these contaminants is unclear, as little has been quantified about children’s play activities in beach and tidal areas affected by contamination. Activities of importance include child contact frequency and duration with media (air, soil, water) containing Oil Spill Chemicals (OSCs), for example, to understand the magnitude of exposures. Short term, intermediate and lifetime exposures also rely on knowing more about the number of visits made to the beach per year, and the time period for those specific health assessments. Health risk calculations with contaminants use exposure data to look at loading on skin, uptake mechanisms and absorption of contaminants into the body and the toxicological impact on cells and organs for non-cancer and cancer endpoints.

Activity patterns of various formats have long been used to study many aspects of society. The functioning, behavior and movement of individuals, communities, and societies for physical activity and health is just one example [3,4,5]. Within the psychology field, activity patterns have been used to understand human behavior interactions how these interactions affect the mind and growth and development [6]. Activity patterns have also been used to understand consumer spending for marketing purposes and societal contributions to the economy [7]. For children, activity patterns are important factors in risk analysis, where the details and type of activity patterns will vary depending on the route of exposure (i.e., contact and therefore uptake through ingestion, inhalation, and dermal routes), and the algorithmic and modeling approach for estimation.

Macro, meso, and micro activity formats have been classified with varying levels of details for use in risk assessment where frequencies and durations of time spent in various locations, engaged in various activities and touching various surfaces are useful for assessing the magnitude of exposures [8]. Macro activities address overall duration engaged in an activity for the day or spent in one location, meso may evaluate time spent within segments of time, and micro-level assesses sequential time in seconds. These various activity patterns can be gathered through observations and note-taking methods (e.g., diary recall), through survey completion and also through videotaping and video-translation methodologies, where one type of collection method may be more suitable or reliable than others for collecting macro, meso, or micro-activity patterns [8].

The Consolidated Human Activity Database (CHAD) is an older and recognized collection of activity patterns [9] used for multiple purposes (predominantly exposure and risk assessment) by researchers and pulls information from 22 studies from 1982 to 2010, where different methods were used to collect the data. CHAD primarily focuses on time spent in various locations and engaged in various activities for both children and adults, where this information can be explored across demographic parameters. Primary modes of activity pattern collection within the CHAD are through observation, diary and survey collection. The National Human Activity Pattern Survey is one of the largest individual studies referenced in the CHAD to look at human behavior relevant to indoor and outdoor exposure and covers a two-year period for 1992 to 1994. Through phone calling, and 24-h diary recall methods, 9384 respondents were queried on time spent in various locations along with other demographic data [10].

Researchers are consistently exploring new and improved methods to collect time–activity data. Traditional pen and paper diary collection methods, for example, have more recently been replaced by smartphone and web applications for periodic or daily entry by participants of relevant study activities [11]. These instrumental approaches assist with shorter and more reliable recall periods improving accuracy of the activity data. GPS tracking has also emerged as a valuable tool to monitor humans in occupational and environmental health studies but typically has been ineffective due to inconsistencies as well as declining accuracy in terms of location. In a recent study performed by Wan et al. (2016) [12], a fuzzy classification scheme was investigated to examine GPS data collected through smartphones to differentiate existing human activity patterns, where an additional aggregation method improved accuracy.

Videotaping and videotranslation methods for the collection of children’s activity patterns were first introduced in 1997 and, although they are not new, they are ideal for collecting sequential contact patterns for dermal exposure of exposed body parts and can be used to evaluate the process of loading and removal at the skin surface [8,13]. The videotranslation component involves the use of a computer software program to maintain the sequential pattern of activity and allow for second time intervals. Zartarian et al. (1995, 1997 [13,14], by utilizing 33 h of videotaped data for four young children, were able to refine the methodologies and summarized right, left-hand and mouth activity in terms of frequency and duration of contact, concluding that these methodologies are an essential means for the collection of activity details for dermal exposure. Videotaping and video translation methodologies were later used to study 23 young children of farmworkers and to assess exposures to pesticides through quantification of their micro-activity patterns [15,16]. In addition, 38 suburban children aged 1 to 6 years were videotaped, and authors were able to obtain micro-level-activity–time-series data (MLATS) to reveal the commonality of short contacts (<5 s) in residential outdoor settings with objects, surface, and hand contact differences by gender, location, and age among participants [17].

Videotaping and videotaping methodologies are also useful for looking at cross-contamination of foods during meal events for non-dietary ingestion exposure [18]. Using the same set of children 38 suburban children previously videotaped, Auyeung et al. (2005) [19], by looking at mouthing behavior, reported that girls had notably a shorter duration of hand to mouth contact as compared to boys (*p* = 0.04), while also mentioning that girls’ frequency of mouthing contact with non-dietary objects as well as hands was higher as compared to that of boys (*p* = 0.008 and *p* = 0.01). Lastly, Auyeung et al. (2005) [19] also described the presence of longer hand contact durations for children who are older than 24 months in contrast to children aged 24 months or less (*p* = 0.04). Some studies have combined videotaping with an observation and hand noting method to obtain frequency count information as opposed to the sequential data that can be provided by videotranslation [20]. Although videotaping methods have been used for activity pattern analysis for use in health risk studies, the number and variety of studies in various settings and for various exposure scenarios is limited. Observations from videos are also widely used for security purposes or even surveillance, occupational exposure, and safety studies [21].

The beach environment is a common space where adults and children recreate. Ashbullby et al. (2013) [22] reported numerous benefits of beaches as health promotion and enhancement tools. The Ashbullby study included both parents as well as children between the ages of 8 and 11 years and demonstrated various physical, social, as well as psychological health benefits, such as enhanced stress relief because of beach visits. Some risks may however present themselves in the beach environment and are worthy of study. Campbell et al. (2019) [23], for example, investigated the extensive effects that anthropogenic beach litter may induce on human health as a result of increased risk of injury in New Zealand. Microbial risks may also be present in sand and water and present exposure concerns for children and adults [24,25]. Oil Spill Chemicals (OSC) may also induce health effects when OSC exposures are high and contact is prolonged.

The study described in this paper is part of the Beach Exposure and Child Health Study (BEACHES) project, which focused on estimating health risk for children to OSCs on the beach following the 2010 Deepwater Horizon Oil Spill in Gulf of Mexico. BEACHES aimed to collect activity patterns for children via videotaping, survey instruments [26] and other field activities [27], estimate oil spill concentrations in the nearshore environment [28,29], and estimate exposure and risk for young children. This paper presents the activity patterns for 120 children collected via videotaping and videotranslation methods to estimate the time spent in microenvironments and engaged in various activities, along with duration of contact with surfaces and objects. These activity patterns are useful in providing the detailed data needed for inhalation, ingestion and dermal exposure estimates, and will be the first time these methodologies have been applied in this setting and for a large sample of children where this level of detail in seconds is provided on activities. The BEACHES project combined activity patterns (i.e., adherence measures, contact patterns) and concentrations in various media (using average or variable concentrations over time) into algorithms to estimate exposures to children to OSCs. This type of micro-activity is particularly useful for the dermal route where sequential contact data files can also be utilized to more closely address loading and off-loading patterns (from water to playing with sand or toys covered with less sand) at the skin surface that might drive diffusion through the skin of a contaminant. Contact rates and therefore exposures to different body parts (left hand versus right hand) were combined for complete exposure or represent averages in populations (left-handed individuals versus right-hand individuals).

## 2. Methods

The project received Institutional Review Board (IRB) approval through the University of Miami (IRB 20140140-MOD00023226) and the University of Texas (IRB #HSC-SPH-18-0396) to study activity patterns of children at beaches and address potential exposures to oil spill chemicals. North Carolina Agricultural and Technical State University fell under the University of Miami IRB.

### 2.1. Project Description and Recruitment

There were 125 children recruited in Miami and Texas through daycare centers, groups for mothers, university contacts, and doctor offices to participate in a field study at four beaches. Participation involved completion of hand press trials and body rinses to quantify soil adherence to hands and body [1,30,31], participation in an approximately one-hour videotaping session, and completion of a parental survey on visits to beaches, children’s observed activities, and risk perception [26]. Additional field data were collected at the beaches to include other child factors (e.g., clothing, toys on the beach, use of sunscreen), and environmental data (e.g., soil condition and weather conditions). There were 120 children that participated in the videotaping section, where factors such as weather hindered some children from completing adequately this part of the field study.

### 2.2. Videotaping Process

Researchers (i.e., videotapers) were trained on the use of the camera (Sony HandyCam FDR-AX33, Sony, Tokyo, Japan) to ensure they understood the controls, were able to focus on the children, and were able to keep the cameras steady during recording. To facilitate steadiness, videotapers were usually seated while videotaping. Videotapers were instructed to stay at least 10 feet away from the children and to utilize zoom lenses to allow visibility. Each child was assigned a team of two, one videotaper and one notetaker. The notetaker collected field data about the activities of the child. The notetaker also helped the videotaper with the camera in terms of moving chairs and fetching water to stay hydrated and stay focused on the activity of the child. Field notes included any items contacted by the child, where potentially the camera would not capture (e.g., the child turned their backs). To collect field notes, the notetaker could move for a better view without coming too close to or distracting the child. Field notes also included start and stop times, cameras used, and disks used. Two teams were always available during the study allowing for up to two children to participate in the study simultaneously. As much as possible, videotaping teams stayed separated to minimize interference between participants.

Before beach videotaping session, study-related documents were sent by email to the guardians and the project was explained by telephone. The guardians provided verbal consent prior to arrival at the beach for those children who opted to continue in the study. Upon arrival at the beach, the team briefly spoke to the parents to inform them again of the expected one-hour videotaping sessions. Parents were advised they could ask the camera to be stopped at any time if they felt uncomfortable, and researchers were instructed to turn cameras off during diaper changes or if children were not wearing clothing. Parents were instructed to play naturally with their child and to ignore the camera. Following the hour of play, parents were then informed the videotaping session was over, and they could bring the child to a field station for pictures and body pool rinses as a part of other field study activities on the project. Each night, two copies of video disks were made for data storage protection.

Some challenges encountered included: crowded beaches, rain or storm events, and overheating for researchers. As much as possible, videotaping teams tried to move to obtain a clear line of sight to the child when beaches became crowded without being disruptive or drawing attention. Crowding of the beach tended to happen in early afternoons. Natural play in a typical beach environment was the aim of the study. Safety was essential and any signs of storms meant parents were advised that videotaping would be halted and resumed later. Researchers watched for storm predictions and called parents ahead of time to cancel when necessary.

### 2.3. Videotranslation

**Figure 1:**
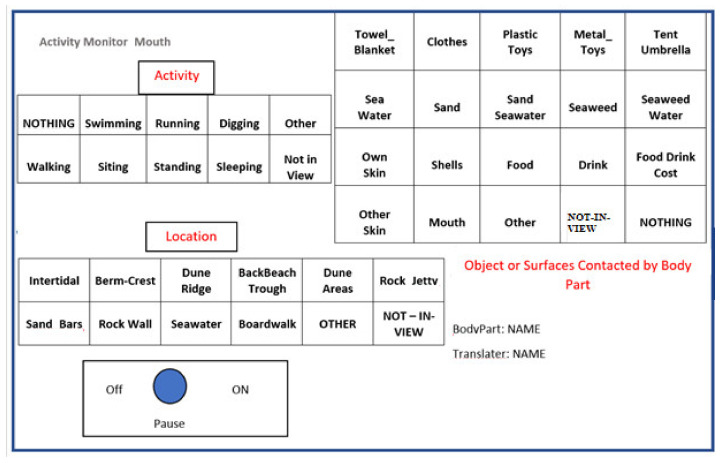
Virtual Timing Device Palette Used for Translation of Location, Activity, and Surface Contacts.

A virtual timing device (a VTD called VideoTraq, Stanford University, Stanford, CA, USA) was used to quantify real-time, sequential micro-activity pattern data collected from the videotapes. VideoTraq has an on-screen window containing three grids, each comprising cells labeled with names of (a) micro-environments (e.g., sand zones, seawater, and other beach zones), (b) activity level (e.g., running, swimming, digging), and (c) potential contact surfaces and objects (e.g., sand, plastic toys, seaweed). To describe the process of translation, while monitoring a contact boundary (e.g., Right-Hand, Left-Hand, Mouth) on the videotape, the VTD translator collected data by activating (i.e., positioning the cursor over a cell and clicking the pointing device) the appropriate cell in each grid (Figure 1). The software couples grid activation with a computer clock that records (seconds), the total duration of each contact event in a text file.

**Figure 2:**
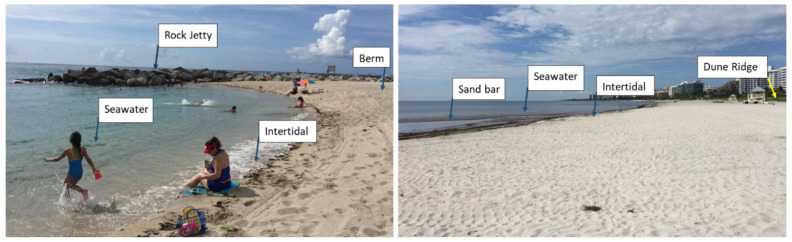
Profile of Haulover Beach (**left**) and Crandon Park Beach (**right**) to Demonstrate Beach Micro-Locations.

Researchers as a team discussed the most common and distinguishable grid names to include on the palette to represent micro Locations, types of Activities, and Surfaces in the beach environment. Some Surfaces were combined, where it was possible and very likely for two surfaces to be contacted at the same time (e.g., Sand–Seawater). For micro-locations, specifically pictures of beaches were collected from various angles and common areas were labelled and defined across the four beaches. *As a caution in reading the results of this research, Seawater is used both for a micro-Location and Surface contacted*. Figure 2 is an example demonstrating the various areas of beaches, where each beach area such as a Berm (or Berm Crest), may vary in width and grade. Other micro-locations such as Boardwalks may exist, and at some beaches, some areas are nonexistent. Seawater is found nearshore and in the Intertidal area. The Dune Area occurs after the Dune Ridge. Researchers also came to agreement using collected videos on the common Surfaces (where Surface also indicate objects such as Plastic Toys) in the beach areas, and the common activities of children. 

The VTD output files allow for detailed analysis and summary information about contact frequency and duration between a child’s body parts and potential surfaces that might be contaminated with OSCs. Moreover, because the data collected were sequential, i.e., each line in the text file corresponds to a real-time activity in the videotape, data were used to reconstruct a child’s activity sequence. Although it was possible to collect three sets of information at a time (location, activity and surface contacts) for this project, four runs through the videotapes were made for each child to capture: (1) Location, (2) Activity, (3) Surfaces contacted for the Right-Hand, and (4) Surface contacted for both the Left-Hand and Mouth. These four separate translations were performed to improve accuracy. Surface contacts for the Left-Hand and Mouth were translated together due to expected limited activities for these body parts and a modified palette was used with two grids for surfaces and objects contacted by the Left-Hand and by Mouth. Previous studies have demonstrated that the left hand is less active than the right hand for most young children. In addition, these studies also indicate that unless children are involved in a continuous eating event, the mouth is also less active [13,14,15,16,17,18,19]. The Mouth grid within the palette is only slightly different to the Left and Right-Hand grid and includes contact with hands. If a grid was not being used to collect information, it was set on “Nothing” and later removed during data analysis.

For this project, there were two main translators and an experienced lead researcher who trained the main translators and who also served as back up on the translation process. Training for the main translators included the following four steps: (1) reading the VTD manual, (2) practicing writing out palettes and grid names to increase familiarity (3) translating 4–15 min video clips with increasing complexity to compare with the results from a more experienced video-translator, called the lead translator. Two trainings were focused on translations for Activity and two were focused on Surfaces contacted. Agreement had to be 90% to move onto each video in the series. Once training was completed, sets of tapes were assigned to translators. Before recording each tape, translators reviewed tapes to become familiar with the child and beach area. To also ensure quality of translation through the process, inter-observer agreement (also known as spot checks), were conducted between translators, where for every ten files translated, the first 15 min of one randomly selected tape was chosen by the lead researcher to be translated by the other translator. Disagreement in percent time allocated to each Activity, Location, or Surface contacted by Right-Hand, Left-Hand or Mouth meant a redo of the entire tape and a discussion on why the disagreement occurred and whether this disagreement occurred in any other tapes in that set of ten. A few decision criteria were established before translations began. One criterion included that for any combination of Activities, Location or Surfaces contacted that occurred at the same time, translators had to choose the category that had the potential to increase exposures, and this was discussed as Sand, Seawater, and Seaweed. Some combinations of surfaces contacted are already present on the palette (e.g., Seaweed and Seawater). Another criterion allowed translators to make intuitive assumptions of where the child was located, even if temporarily out of view. This assumption could be made when there was a high chance the child had remained in the same location and was blocked by someone. Translators also discussed other categories of potential Surfaces or Activities not included in the grid and decided on grid names that would be best suited for those, in addition to when to use the Not-In-View, Nothing and Other selections on the grids.

### 2.4. Spot Checks to look at Interobserver Agreement Over Body of Translations

For every 10 segments translated, the two videotranslators cross translated 15 min of each other’s segments as randomly indicated by the lead experienced researcher. If the agreement was under 90%, that entire segment was re-translated, following discussions on Surface, Location, or Activity classification and designation by the entire research team.

### 2.5. Analysis and Statistical Methods

A total of 120 children’s videos were translated in order to evaluate their Activities, Locations, and Surface contacts for Right-Hand and Left-Hand/Mouth, resulting in four separate text files of sequential activities in seconds. These files were imported into MS Excel to create one workbook with worksheets (or tabs) for each child. To validate the merging, tables were manually curated and checked for the consistency of record and subsequently validated using SAS script looking for total time recorded for each child. Because all videos were translated independently for body parts, there were slight deviations in total time. We adjusted the time by finding the minimum time of translation for the four files for each child and truncated the other files to that total time, for subsequent analysis and overlapping of data, ensuring start times were the same. These data tables were then imported into the SAS system (SAS Enterprise Guide Version 7.13, Cary, NC, USA) by creating a database for further processing and analysis.

Given that four translations were completed per video, additional analyses included overlapping data by comparing data across translations. For example, since one translation focused on location while another focused on surface contacted, translations were compared to determine the duration of time that children spent contacting a surface while in a particular location. Tables generated from each translation were overlapped, using a structured query language (SQL) procedure, by searching for the overlapping time where children were engaged in categories of activities. All data were normalized by the total time videotaped per child, for averages and variances based on percent time engaged in an activity where some children were videotaped for more than one hour and some less, with the range of time being 15 min–81.2 min (average = 61.7 min).

The SAS means procedure was used to calculate descriptive statistics for categories of Activity, Location, Right-Hand, Left-Hand, and Mouth. The MEAN procedure was also used to generate statistics for the children grouped by their Sex (Male/Female), Age Groups 1–3 (i.e., 1 to 24 months, 25 to 48 months and >48 months) and by Beach (Crandon, Haulover, Seawall, and Stewart). Photos of each beach are provided in Supplementary Materials Figure S1 for reference. For comparisons of the mean time spent in various locations or on various activities across multiple age groups or beach locations, ANOVA tests were carried out to test the null hypothesis that all group means were equal within the location or activity or surface. If this test was rejected, individual means were compared using Tukey’s multiple comparison test. *T*-Tests were used to look at differences across sex. Associations and findings were considered significant at a *p* < 0.05 alpha level and moderately significant at *p* < 0.10 alpha level, where these findings are highlighted. For the Tukey multiple comparison test, adjusted *p* values are reported between groups regarding significance.

## 3. Results

**Figure 3:**
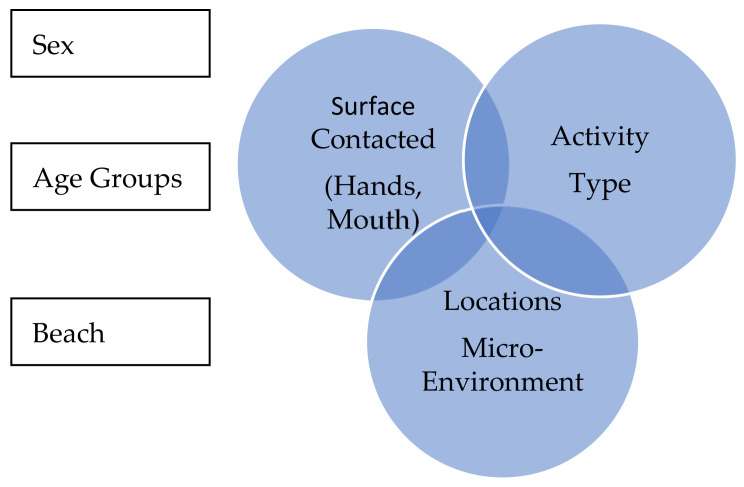
Overlapping Location, Activity and Surface Contacted to examine Trends.

Results were first explored by Location and Activity across all children. Location and Activity were then explored by Sex, Age Group and by Beach to look at any differences. Surfaces contacted were evaluated across all children, and then by Sex, Age Group, and Beach. Overlapping data across Location, Activity, and Surfaces contacted are presented for all the children and then again by Sex, Age Groups, and Beaches, to examine relationships and trends (Figure 3).

For reference and to better understand the distribution and analysis of data, Table 1 depicts the distribution of children by age and sex at the four beaches, whereas Table 2 shows the locations that were present at each beach and that any child visited.

### 3.1. Location and Activity Across all Children

**Figure 4:**
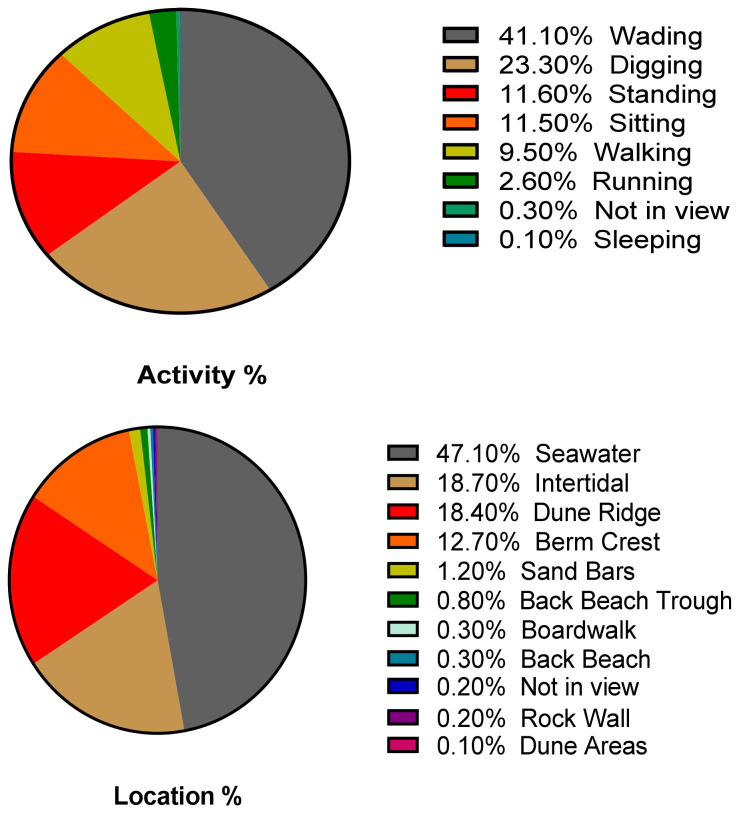
Percentage of Time Engaged In Various Activities and in Various Locations for All Children (*n* = 120).

The Location and Activity patterns of the 120 children are shown in Figure 4 (details in Supplementary Materials Table S1). The greatest amount of time was spent on the Activity of Wading (41.1%) and then Digging (23.3%). The least amount of time was spent Sleeping (0.10%). For Location, children spent the greatest amount of time in the Seawater (47.1%), Intertidal Zone (18.70%), and Dune Ridge (18.4%) (Figure 1, details in Supplementary Materials Table S1). The least amount of time was spent in the Dune Areas (0.10%).

### 3.2. Location and Activity by Sex

We explored whether Activity and Location varied by Sex in the beach environment. The majority of time was time spent Wading for both male (40.7%) and female (41.5%) children (Table 3). This was followed by Digging for female (23.2%) and male (22.9%) children. There were some moderately significant differences between males and females where boys spent a little more time Running 3.1% vs. 2.1% (*p* = 0.066). The children spent very little time Swimming and Sleeping. Regarding Location, most of the time was spent in Seawater by both female (45.8%) and male (47.5%) children. This was followed by the Intertidal Zone, which was the second most visited location by females (20.3%) and males (16.9%) (Table 1). Girls spent more time on Sand Bars than boys, 2.1% versus 0.1% (*p* = 0.075), where Sand Bars were only found at Crandon beach. The greatest time spent in Seawater coincides with the greatest time spent Wading as an Activity. Table 3 reports *p* values for differences in male as compared to female in addition to combined (male plus female) 95 percent confidence intervals for time spent on various activities and in different locations.

### 3.3. Activity and Location by Age−Groups

The Activities children engaged in and the Locations they explored were analyzed by Age Group. Age Group 1 refers to 0 to 24 months, Age Group 2 refers to 25 to 48 months and Age Group 3 refers to children greater than 48 months. The greatest time spent was Wading and was 38.9% for Age Group 1, 37.6% for Age Group 2, and 45.1% for Age Group 3 (Table 4). The mean time spent on the Activities of Sitting, Digging, and Running by the youngest children was significantly different from the other two age groups (*p* < 0.05). Not surprisingly, the older children spent more time digging and running and less time sitting. For Location, the greatest time was spent in Seawater by all three Age Groups (45.4%, 43.7%, and 49.5%, respectively for ascending age groups, youngest to oldest). The other two Locations where all three groups of children spent most of their time were in the Intertidal Zone and Dune Ridge. There are some Activities and Locations where children of all Age Groups spent little to no time (e.g., Swimming, Sleeping, Rock Jetty and Dune Areas). Interestingly, for sleeping, there were two older children (70 and 76 months) and one young child (45 months) that spent some brief time asleep.

### 3.4. Activity and Location by Beach

The time spent on various Activities and in various Locations was explored by Beach. There were some significant differences (adjusted *p* < 0.05) across Beaches for Activities and Locations and these are indicated by the “*” symbol in Table 5. As indicated above, results showed that for all four Beaches, on average, children spent most of their time Wading. This was especially the case at Seawall Beach, which had the highest mean time spent on Wading of 54.8% (Table 5). The second most common activity was Digging at all Beaches, with Stewart having the highest mean time spent Digging of 29%. Regarding Location, children at the Crandon beach spent most of their time in the Dune Ridge (38.3%). For Haulover, Seawall and Stewart beaches, children spent most of their time in the Seawater (i.e., 50%, 59.8% and 42% of their time, respectively). Again, there were some Activities and Locations that had low to little time spent on them across all Beaches, such as Swimming and Rock Jetty. Notable is that the least amount of time spent Wading was at Crandon of 28.4%, and coincides with least amount of time spent in Seawater of 32%.

### 3.5. Left-Hand, Right-Hand, and Mouth Surface Contacts Across all Children

**Figure 5:**
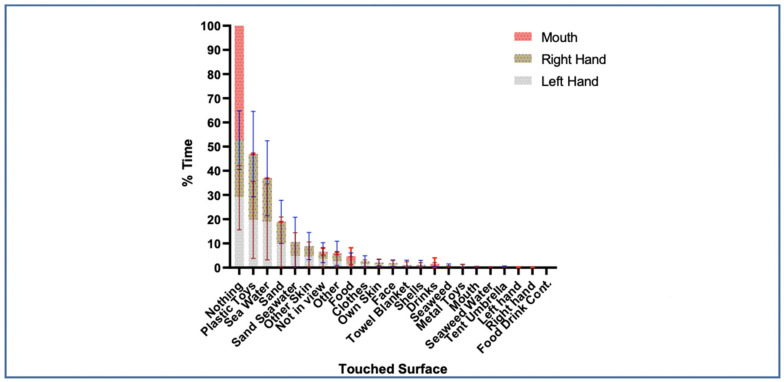
Mean Percent Time Right-Hand, Left-Hand, and Mouth Contact Surfaces.

For the Left-Hand, most of the time, they touched Nothing. For those times when the Left-Hand did come into contact with a surface or object, results indicated that children touched Plastic-Toys most of the time with a mean percent time of 19.7% (Figure 5, Supplementary Materials Table S3). The next most touched Surface for the Left-Hand was Seawater (18.9%) and then Sand (9.8%). For the Right-Hand, Plastic-Toys were touched the most (27.2%), followed by Nothing (23.8%). The next most touched Surface for the Right-Hand was Seawater (18%), Sand (9.1%), and the combination of Sand−Seawater (5.1%). Some Surfaces that were rarely contacted by children to include their Mouth, and their other hands, Umbrella, and Drink and Food Containers (Figure 5). In some cases, standard deviations were high (Supplementary Materials Table S3) indicating high variability among children.

For the majority of the time, the Mouth was not in contact with any Surface. When the Mouth did contact a Surface, the most common item was Food, with an average of 2% of total time. Finally, the next item touched most with the mouth was Drinks (1.2%). Plastic-Toys and Other. The results for the Right-Hand, Left-Hand and Mouth can be found in more detail in Supplementary Materials Table S2.

### 3.6. Left-Hand, Right-Hand and Mouth Surface Contact by Sex, Age Groups, Beaches and Locations 

We found that the most touched surface for hands, after Nothing, remained Plastic-Toys for both female and male children, where for female and male children the percentage of time for the Right-Hand was 26.0% and 18.7%, respectively and for the Left-Hand was 18.8% and 20.8%, respectively. We explored if significant differences existed by Sex and found that for the Left-Hand, the only significant difference was for “Not in View” (*p* = 0.0226) for males (4.4%) and females (2.4%). For the Right-Hand and Mouth, no significant or moderately significant differences in Surface contacts were found between males and females. The results can be found in Supplementary Materials Table S3.

There was a significant difference (adjusted *p* < 0.05) for the Left-Hand between Age Group 1 and Age Group 3 for Sand contact (4.9%, and 11.8%, respectively) and Seawater 0) of 14.0% and 21.7%, respectively. For Surface category of Other-Skin, there were significant differences between Age Groups 1 and 2, 1 and 3, and 2 and 3 (*p* < 0.05), where the youngest children were in contact with the skin of their parent, guardian or sibling. For the Right-Hand, there were significant differences between Age Groups 1 and 2 and between Age Groups 1 and 3 for contacting Other-Skin. Furthermore, for the Right-Hand, there were significant differences between the Right-Hand touching the Mouth for Age Groups 1 and 3, where only the younger children demonstrated this activity. For the Right-Hand, there were significant differences (*p* < 0.5) for touching Nothing between the Age-Groups 1 and 3 (28.5% versus 22.1%). For the Sand–Seawater combination, there were significant differences between Age Groups 1 and 2 and between Age Groups 1 and 3, where the youngest Age Group contacted this combination the least.

For the Mouth, there were significant differences for Plastic-Toys between Ages Groups 1 and 2 and 1 and 3, where both of the oldest Age Groups touch Plastic-Toys less with the Mouth. Finally, for Mouth contact with Drinks, there were significant differences between age groups 1 and 2 and groups 1 and 3, where Age Group 1, the youngest children seemed to drink more. The results can be found in Supplementary Materials Table S4. Significant differences (adjusted *p* < 0.5) were found for Crandon (Cr), Haulover (Ha), Seawall (Se), Stewart (St) for Surfaces touched by the Hands and Mouth (Supplementary Materials Table S5). 

For the Left Hand we found significant differences between touching Plastic-Toys in Ha–Se (11.7% versus 24.9%) and Ha–St (11.7% versus 22.8%). For Left-Hand touching Seawater by Beach, we found significant differences for Ha–Se (30.6% versus 18.4%), Ha–St (30.6% versus 12.5%), and Cr–Ha (12.1% versus 30.6%). For the Sand/Seawater combination, we also found significant differences for Cr–Se, Cr–St, Ha–Se, Ha–St, where the Texas Beaches of Stewart and Seawall had higher Left-Hand contacts with this combination of 8.6% and 9.8% (Crandon and Haulover had only 0.5% and 0.8%). What is notable is that the Texas beaches had much wider intertidal areas, enticing children to play in the area where both Sand and Seawater are found for increased contact. Interestingly, for Not-In-View, the Cr–Ha, Ha–St, Ha–Se Beach comparisons had significant differences where Haulover was the highest with 7.2%, Crandon was 3.9%, and Stewart and Seawall were 1.1% and 1.6%, respectively. Haulover was a very busy beach with angled slopes and had a tighter area for videotaping and explains the need for the translator to use the Not-In-View button more for contact of the Left-Hand. For Drinks, Cr–Ha, and Cr–Se had significant differences, where at Crandon, the children drank more of 1.3% of the time compared to Haulover of 0.1% and Stewart of 0.5%. Drinking (represented in the Left-Hand holding a drink) more could be a reflection of hotter or more humid videotaped days. Hotter or more humid days might also influence the time children spend in the Seawater cooling off. Details about average environmental conditions at each beach during the study period is available in the supplemental text of [30].

For the Right-Hand contacts by Beaches, significant differences for Plastic-Toys were found between Ha and St (18.6% versus 33.6%). Similar to Left-Hand, for Right-Hand with Seawater, we found differences between Cr and Ha (13.7% versus 30.9%), Ha and Se (30.9% versus 14.7%), Ha and St (30.9% versus 10.8%). Haulover had the lowest turbidity and thus most inviting clear water for swimming and wading. For the Sand–Seawater combination, we found differences between Cr and Se (0.7% versus 11.1%), Cr and St (0.7% versus 10.4%), Ha and Se (0.7% versus 11.1%), St and Ha (10.4% versus 0.7%), again given the wider Intertidal areas at Texas Beaches. For Not-In-View, there were some significant differences between Cr and Se, Cr and Ha, Ha and St, Ha and Se, and Cr and St, with Haulover having the largest percent time of 5.9%. For Food, Ha–Se had significant differences (1.8% versus 0.1%). For Own-Skin contact with the Right-Hand, Cr–St, Ha–St, Se–St were all significantly different, although these contacts were all low percent contacts. Face contacts were significantly different for Ha–Se. Shells contacts were significantly different for Cr–Ha, Ha–St. And lastly Seaweed contacts were different for Cr–St, Ha–St, Se–St. Face, Shells and Seaweed contacts were all low (all <1.5%).

For the Mouth, we only found significant differences for Food between Ha and Se (3.5% versus 0.8%), where again the Mouth was most of the time in contact with Nothing. The details on these contacts by Beaches can be found in Supplementary Materials Table S5. Other details for Left-Hand, Right-Hand and Mouth Contacts while engaged in Activities can be found in Supplementary Materials Table S6. In addition, surface contacts by Left Hand, Right-Hand and Mouth while in Locations can be found in Supplementary Materials Table S7.

## 4. Discussion

This study explored children’s activity patterns at four Beaches to understand where children spend the most time in the various Locations of the beach (e.g., Seawater, Intertidal, Dune Ridge), what Activity they engaged in the most (e.g., Running, Digging), and what Surfaces they would typically contact (e.g., Plastic-Toys, Sand). These activity patterns were presented by average mean percent normalized by videotaping time across all 120 children and explored for differences by Sex, Age Groups, and by Beaches, and overlapped to look at inter-relationships. Some interesting findings can be related to determining exposure and risk to contaminants across Age Groups, Sex and Beach areas.

Mean percent time spent in Locations were highly influenced by profile and even appearance found at each Beach, where this also, in turn, had an impact on time spent in an Activity. We found that children were highly involved in the Activity of Wading (41.5%). The oldest Age Group engaged in this activity slightly more (45.1%) as compared to the younger age groups (38.9%, and 37.6%). Wading was also highest for Seawall (54.8%) and lowest for Crandon (28.4%), where this was likely influenced by the substantial presence of seaweed on the sands and seawater at Crandon. As expected, Wading was the most observed Activity in the Intertidal Zone and Seawater. Seawater is also where children spent the most time compared to other Locations. Females and males had very similar average mean percent times spent in Locations and Activities. For Location, the only significant differences found indicated that females spent more time on Sand Bars at Crandon Beach. Although not significant, females seem to spend more time in the Intertidal area and males spent more time in the Dune Ridge. Again, Wading is common in the Intertidal zone and Digging is more common in the Dune Ridge.

There are some Activities and Locations where children of all Age Groups spent little to no time (e.g., Swimming, Sleeping, Rock Jetty and Dune Areas). For locations, this was partly due to that location only being found in one Beach location, for example, Sand Bars were only found at the Crandon Beach and Rock Jetty was only found at the Hanover Beach. It is also evident that families spent more time closer to the Seawater, although children might occasionally explore the back areas of Beaches and in particular for boys running or chasing balls (i.e., observed for some kids and designated as Plastic Toys). Swimming and Sleeping were also less common Activities, where swimming is more challenging in a Seawater environment and Sleeping is unlikely for such short observed time periods, where children and family intentionally go to engage in active play. We do see where children contacted the combination of Sand−Seawater most at the Texas Beaches of Stewart and Seawall which had long Intertidal areas. Likewise, we see where children spent a significant time in Seawater at Haulover, a beach which all researchers agreed had the clearest water and least amount of seaweed. For difference by Sex, we found that males and females behaved in a very similar fashion, however males liked to run a little more than females. Notable is the least amount of time spent Wading at Crandon of 28.4%, and this coincides with least amount of time spent in Seawater of 32% and likely due to the significant amounts of seaweed on this beach in the Intertidal zone and Seawater areas and likely a deterrent to children and the adults that supervised them. This also coincides with the least amount of time spent in the Seawater of 32% mean percent time.

Not surprisingly, the older children spent more time Digging and Running and less time Sitting than the younger children, where these differences in activity level are important considerations in higher breathing rates to use for risk assessment calculations. Likewise, we see that children liked spending time in Seawater the most, and this is one of the main entertainments and reasons families go to the beach. Seawater is therefore an important consideration when evaluating the potential risks from contaminants.

For the youngest age group of children, not surprisingly, we see that they drink and eat more and also contacted the skin of their parent/guardian or older sibling in order to better navigate the beach area. Many times, they were carried by their parent, and so their activities might be more driven by what the parents decide would be more entertaining for their child.

For food contacted by Hands or Mouth, this can be influenced by the amount of food brought to the beaches. Stewart had a big building with bathrooms and shops and some families were eating at this building. Crandon also had a concession stand located some distance from the beach. Seawall and Haulover did not have eating facilities. Under normal circumstances, the presence of these facilities might influence families to bring less food, and children may not snack as often. However, knowing that they are involved in a study, may have influenced most families to bring food more consistently across all beaches.

When it comes to contacts, some results are expected, and some contacts are worth revisiting. Children liked to play with Plastic-Toys, which were the most touched Surface. These plastic toys included buckets, shovels and balls as observed on camera. This is not surprising; many brought these toys to the Beach. In residential studies, children are observed to play with many toys as expected, however the names of grid cells (i.e., selections on the Videotranslation Palette) are still difficult to compare. In these studies, surfaces were indicated as metal or plastic, and more to depict the ability of the contaminant to absorb into a surface or remain on a surface [17,19]. Haulover was the beach where children touched Plastics-Toys the least. This could have occurred for two reasons; they carried less toys to this beach or because the water was so inviting, they spent more time swimming. Seawater was the second most touched surface and coincides with spending significant time in the water. Sand was most contacted surface by Right-Hand and Left-Hand in the Intertidal area (18.2%, 18.3%), and on Sand Bars (23.3%, 12.8%), and was high also in other micro−locations. Any review of Sand contact should take into account the combination of Sand−Seawater. There are some observable differences also in the more popular Surfaces contacts by the three Age Groups. The oldest Age Groups touched Seawater more, because they were in the Seawater, Wading more. The youngest Age Group spent more time contacting Plastic-Toys, where spending more time Sitting allowed for more sand play with toys.

Roughly 90% of humans are right-handed, with children generally showing varying hand preference trends and are commonly slower with both hands [32]. In addition, performance differences between the hands are significantly different for younger children but improves with age. Research has indicated that hand preference can be reliably detected from six months of age onward [33]. However, variable hand use preferences such as fluctuating from left to right hand has been noted in infancy, demonstrating the malleability of hand preference during infancy [34] and different patterns of development [35]. These studies though have tended to evaluate children older than the children observed in this study [36] but offer critical insight into the behavioral patterns observed. In this study, the Right-Hand was more active than the Left-Hand in contacting objects, where while digging, for example, the Right-Hand handled the tool (i.e., Plastic-Toy shovel) more while the Left-Hand often contacted the Sand.

## 5. Conclusions

This is the first study of its kind to collect very detailed activities for children at beaches, making it difficult to compare to other studies in the field. Videotaping studies have commonly been conducted in and around indoor and outdoor residential areas and, in some cases, in suburban and agricultural areas [15,17,19]. The micro-locations in these studies were different. The locations in these residential studies were concerned with rooms of the home and outdoor areas (e.g., living room, bedroom, patio, yard) [15,17,19], where researchers believe contaminant concentrations may vary. In a similar manner in this beach study, contaminants may vary in the Intertidal zone of the beach compared to Seawater.

Limitations of this study include the short videotaping time, although substantial data were collected over 120 children providing for an assessment of differences across ages, sex, and beach areas. Although there is great variability for some behaviors, trends by age groups and beach profiles are evident. The presence of the camera can also cause interference; however, the research team took great effort to use the zoom lens, be discreet, and reduce distractions. Because children were being videotaped in the natural environment, interference from other beachgoers may have occurred and in some cases resulted in some time designated as Not-In-View. Not-In-View was slightly greater when quantifying surface contacts. Translators could more often discern Location or Activity more readily. Use of Not-In-View was also affected by the lack of a 360-degree view of the children, given the ocean on one side. Some errors may still occur in the data during translation but were limited as much as possible through training, interobserver agreement checks, and retranslation where deemed necessary.

This study offers a wealth of data. Various approaches can be used to study, mine and utilize the data. Contact frequencies and sequential data, for example, can be quantified and used in risk assessment algorithms to address the dynamics of loading at the skin surface. This research is supplemented by previously published survey data that include macro-activity, such as the number of visits to beach and time typically spent [26] and adherence of sands to the hands and body of children [30,37]. Future work will compare micro-activity field with survey data on children’s activity patterns that were previously published, where parents relied on recall to advise how their children like to spend time (in Seawater versus Intertidal zones) [26]. Weather data and evenly collected soil temp and soil texture from the videotaped days and from beaches will also be looked at to determine how the weather and condition of soil might have influenced where children preferred to spend time (e.g., Seawater instead of Dune Ridge) and what might have influenced some activities (e.g., Drinking more).

Although activity pattern collection through videotaping and video-translation can be labor intensive, once collected, they can be widely useful for estimates of exposures to other contaminants in the beach environment (e.g., microorganisms) and to other contaminants and environments where children can be exposed [38]. Micro, meso and macro-activities can be extracted and used to explore inhalation, ingestion and dermal exposures. Data collected may be also of interest for social science studies that focus on how families spend time and engage in their surroundings for social interaction. The beach is an important environment for children’s play. In a University of California, Los Angeles study, individuals at 11 beaches were surveyed (1146 surveys), and researchers found that 31% visited the beach for a place for children to play, where a common activity desired was wading [39]. It is critical, therefore, to look at hazards in that environment and to understand how children play and how exposure might occur.

A previously published paper published by this group looked at addressing data gaps in being able to determine health risks to adults and children from oils spills and the resulting contaminants’ concentration in media [1]. Our findings show that researchers should focus on determining contaminants found in shallow areas of seawater and the intertidal regions and also in sand areas close to the water where children spent the most time. In addition, based on activity patterns, exposures for young females and males may be similar, where for dermal exposure and ingestion exposure, the right hand is influential. Our study also showed that the profile and appearance of the beach influences where children spend time, and these factors should be considered in risk assessment estimates.

## Figures and Tables

**Table 1:** Number, Sex and Age Group of Children by Beach.

Age Groups (Months)	Crandon	Haulover	Seawall	Stewart
Girls				
1 (0–24)	3	4	3	5
2 (25–48)	4	9	2	4
3 (>48)	6	7	9	11
Boys				
1 (0–24)	4	3	5	0
2 (25–48)	5	7	6	4
3 (>48)	5	3	6	5

**Table 2:** Presence of Locations at Beaches.

Beach Location	Crandon	Haulover	Seawall	Stewart
Back Beach	√		√	√
BackBeach Trough			√	√
Berm Crest	√	√	√	√
Boardwalk	√	√		√
Dune Areas	√		√	√
Dune Ridge	√	√	√	√
Intertidal	√	√	√	√
Rock Jetty		√		
Rock Wall	√	√	√	√
Sand Bars	√	√	√	
Seawater	√	√	√	√

√—Yes.

**Table 3:** Mean Percent Time Spent Engaged in Various Activities and Locations for Children by Sex (females, *n* = 67, males, *n* = 53).

Activity/ Location	Variable	Female		Male		*p*-Value	95% CI
		Mean %	Std. Dev	Mean %	Std. Dev		
Activity	Wading	41.5	24.6	40.7	23.1	0.850	36.5–45.5
	Digging	23.2	16.8	22.9	16.6	0.937	20.1–26.1
	Sitting	12.2	14.9	10.5	13.3	0.505	8.89–14.0
	Standing	11.3	10.4	12.5	11.8	0.547	9.85–13.8
	Walking	9.2	6.8	10.1	7.0	0.486	8.32–10.8
	Running	2.1	2.5	3.1	3.4	0.066*	1.98–3.05
	Not in view	0.3	0.8	0.2	0.4	0.414	0.142–0.389
	Sleeping	0.2	1.3	0.0	0.0	0.269	−0.061–0.279
	Swimming	0.0	0.0	0.0	0.1	0.264	−0.003–0.015
	Other	0.0	0.0	0.0	0.0	0.376	−0.0002–0.0006
	Total	100		100			
Location	Seawater	45.8	23.6	47.5	24.5	0.706	42.3–50.9
	Intertidal	20.3	22	16.9	16.1	0.343	15.2–22.3
	Dune Ridge	15.3	22.4	21.6	27.2	0.168	13.6–22.6
	Berm Crest	13.1	21.4	12.6	17.9	0.893	9.34–16.5
	Sand Bars	2.1	7.7	0.1	0.7	0.075 *	0.148–2.26
	Back Beach Trough	1.5	12.1	0.0	0.0	0.378	−0.805–2.46
	Boardwalk	1.3	10.4	0.0	0.2	0.389	−0.681–2.13
	Not in view	0.3	0.7	0.2	0.3	0.242	0.126–0.331
	Rock Wall	0.2	1.2	0.2	1.1	0.892	−0.034–0.376
	Back Beach	0.1	0.4	0.5	1.5	0.027 *	0.112–0.501
	Dune Areas	0.0	0.1	0.3	1.6	0.124	−0.038–0.342
	Rock Jetty	0.002	0.013	0.0	0.0	0.376	−0.0009 –0.0020
	Other	0.0	0.0	0.0	0.0	0.376	−0.0004–0.0012
	Total	100		100			

* *p* values indicate significant or moderately significant difference between female and male children. *p* values represent differences in male as compared to female in addition to combined (male plus female) 95 percent confidence intervals for time spent on various activities and in different locations.

**Table 4:** Mean Percent Time Spent Engaged in Various Activities and Locations by Age Groups, 1–3 (for 0–24 months, *n* = 27, for 24–48 months, *n* = 41, and for >48 months, *n* = 52).

Activity/ Location	Variable	1 (0–24)		2 (25–48)		3 (>48)	
		Mean %	Std. Dev	Mean %	Std. Dev	Mean %	Std. Dev
Activity	Wading	38.9	23.3	37.6	24	45.1	23.8
	Sitting * (1–2), (1–3)	19.9	18.3	10.1	12.9	8.2	11
	Standing	14.4	13	14	12.7	8.8	7.3
	Digging * (1–2), (1–3)	13.0	12.4	26.6	16.6	25.5	16.8
	Walking	12.6	10.6	8.2	5.2	9.1	5
	Running * (1–2), (1–3)	1.0	2.3	3	3.5	2.9	2.5
	Not in view	0.2	0.3	0.4	1.1	0.2	0.3
	Swimming	0.0	0.0	0.0	0.0	0.0	0.0
	Sleeping	0.0	0.0	0.0	0.0	0.2	1.4
	Other	0.0	0.0	0.0	0.0	0.0	0.0
	Total	100		100		100	
Location	Seawater	45.4	22.6	43.7	24.9	49.5	23.9
	Intertidal	21.0	23.1	17.5	17.7	18.6	19.5
	Dune Ridge	18.1	23.1	23	28.4	14.2	22.0
	Berm Crest	14.2	21.5	11.9	20.8	13.1	18.4
	Sand Bars	0.4	1.5	0.7	3.6	2.0	8.2
	Back Beach	0.3	1.0	0.3	1.2	0.3	1.0
	Rock Wall	0.4	1.8	0.2	1.3	0.0	0.1
	Not in view	0.1	0.2	0.4	0.9	0.2	0.3
	Rock Jetty	0.0	0.0	0.0	0.0	0.0	0.0
	Back Beach Trough	0.0	0.0	0.0	0.0	1.9	13.7
	Boardwalk	0.0	0.0	2.1	13.3	0.0	0.0
	Dune Areas	0.0	0.0	0.2	1.3	0.2	1.1
	Other	0.0	0.0	0.0	0.0	0.0	0.0
	Total	100		100		100	

* Indicates significant (adjusted *p* < 0.05) difference between Age Groups in brackets.

**Table 5:** Mean Percent Time Spent Engaged in Various Activities and Locations by Beach

Activity/ Location	Variable	Crandon *n* = 27		Haulover *n* = 33		Seawall *n* = 31		Stewart *n* = 29	
		Mean %	Std. Dev	Mean %	Std. Dev	Mean %	Std. Dev	Mean %	Std. Dev
Activity	Wading * (Cr–Se), Cr–Ha), (Se–St)	28.4	21.8	44.6	22.7	54.8	20.7	34.5	22.4
	Sitting * (Cr–Se), (Ha–Se)	15.9	15.6	14.7	18.7	3.8	4.2	11.8	10.8
	Standing * (Cr–Se), (Ha–Se)	17.0	12.4	14.3	12.0	5.8	5.8	10.7	9.8
	Digging	25.7	17.3	18.3	14.5	20.4	15.7	29.0	17.7
	Walking * (Ha–St), (Ha–Se)	10.5	6.7	5.6	3.8	11.0	6.7	11.7	8.2
	Running * (Ha–Se), (Se–St)	2.2	3.1	1.6	2.2	4.1	3.8	2.1	1.8
	Not in view	0.3	0.4	0.4	1.2	0.1	0.2	0.3	0.4
	Swimming	0.0	0.1	0.0	0.0	0.0	0.0	0.0	0.0
	Sleeping	0.0	0.0	0.4	1.8	0.0	0.0	0.0	0.0
	Other	0.0	0.0	0.0	0.0	0.0	0.0	0.0	0.0
	Total	100		100		100		100	
Location	Seawater * (Cr–Se), (Cr–Ha), (Se–St)	32.0	22.4	50.0	20.9	59.8	19.8	42.0	24.7
	Intertidal * (Cr–St), (Ha–St)	9.8	21.9	15.0	15.0	20.2	15.5	29.8	21.4
	Dune Ridge * (Cr–St), (Cr–Se), (Ha–Se)	38.3	30.9	20.4	22.6	5.7	13.0	9.9	17.4
	Berm Crest	13.2	21.3	13.6	22.7	13.9	16.5	10.9	19.1
	Sand Bars * (Cr–Ha), (Cr–Se), (Cr–St)	5.4	11.6	0.0	0.0	0.0	0.0	0.0	0.0
	Back Beach	0.6	1.5	0.0	0.0	0.3	0.9	0.4	1.4
	Rock Wall	0.0	0.1	0.6	2.1	0.0	0.0	0.0	0.0
	Not in view	0.3	0.5	0.4	0.9	0.0	0.2	0.2	0.4
	Rock Jetty	0.0	0.0	0.0	0.0	0.0	0.0	0.0	0.0
	Back Beach Trough	0.0	0.0	0.0	0.0	0.0	0.1	3.4	18.4
	Boardwalk	0.1	0.1	0.0	0.2	0.0	0.0	2.9	15.8
	Dune Areas	0.3	1.5	0.0	0.0	0.0	0.1	0.3	1.5
	Total	100		100		100		100	

* Indicates significant (adjusted *p* < 0.05) difference between age groups in brackets.

## Supplementary Materials

The following are available online at https://www.mdpi.com/1660-4601/18/6/3274/s1, Figure S1 and Tables S1–S7.
