# Supplementary material for: Quantified Activity Patterns for Young Children in Beach Environments Relevant for Exposure to Contaminants
Source: Int J Environ Res Public Health. 2021 Mar 22;18(6):3274. doi: 10.3390/ijerph18063274 (PMC8004776; doi:10.3390/ijerph18063274)
Supplement: Supplementary file 1 [file ijerph-18-03274-s001.pdf]

Supplemental Information

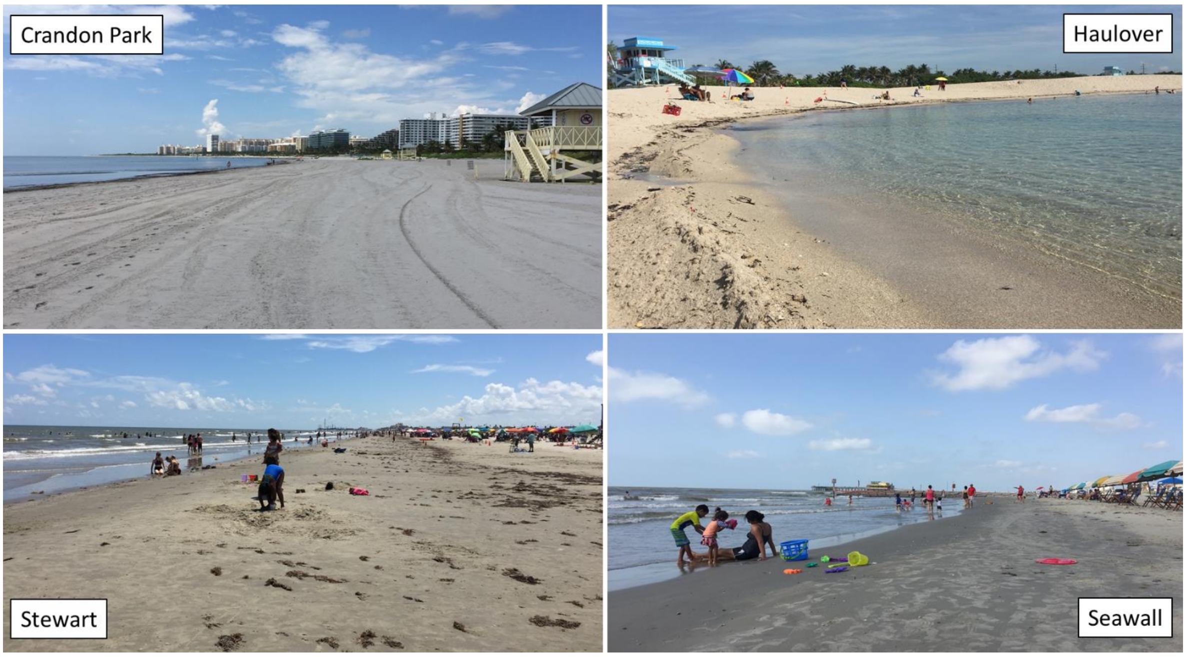

Figure 1. – Photos of Each Beach.

Table 1. Mean Percent Time Engaged in Various Activities and Location for All Children.

	Variable	Mean%	Std. Dev
Activity	Wading	41.2	23.8
	Digging	23.1	16.6
	Standing	11.8	11.0
	Sitting	11.5	14.2
	Walking	9.6	6.9
	Running	2.5	3.0
	Not in view	0.3	0.7
	Sleeping	0.1	0.9
	Swimming	0.0	0.0
	Other	0.0	0.0
Location	Seawater	46.6	23.9
	Intertidal	18.8	19.6
	Dune Ridge	18.1	24.7
	Berm Crest	12.9	19.8
	Sand Bars	1.2	5.9
	Back Beach Trough	0.8	9.0
	Boardwalk	0.7	7.8
	Back Beach	0.3	1.1
	Not in view	0.2	0.6
	Rock Wall	0.2	1.1
	Dune Areas	0.2	1.1
	Rock Jetty	0.0	0.0
	Other	0.0	0.0

Table 2. Mean Percent Time Left-Hand, Right-Hand, and Mouth Surface Contacts.

Surface	Left-Hand		Right-Hand		Mouth	
	Mean%	Std. Dev	Mean%	Std. Dev	Mean%	Std. Dev
Nothing	28.9	13.3	23.8	12.1	96.1	5.1
Plastic Toys	19.7	15.9	27.2	17.7	0.1	0.3
Sea Water	18.9	15.7	18.0	15.5	0.0	0.1
Sand	9.8	11.1	9.1	8.9	0.0	0.1
Sand Seawater	4.9	9.5	5.7	10.2	0.0	0.0
Other Skin	4.7	5.9	4.2	5.6	0.0	0.0
Not in view	3.5	4.8	2.7	4.1	0.4	1.4
Other	2.6	3.7	3.3	5.0	0.1	0.6
Food	1.3	3.2	1.4	3.4	2.0	3.5
Clothes	1.3	1.9	1.3	2.3	0.0	0.0
Own Skin	1.3	2.2	0.8	1.3	0.0	0.0
Face	1.1	1.8	0.8	1.3	0.0	0.0
Towel Blanket	0.5	1.9	0.5	2.1	0.0	0.0
Shells	0.5	1.6	0.6	1.9	0.0	0.0
Drinks	0.4	1.7	0.0	0.1	1.2	2.4
Seaweed	0.1	0.7	0.3	1.1	0.0	0.0
Metal Toys	0.1	1.3	0.1	1.1	0.0	0.0
Mouth	0.1	0.5	0.0	0.2	0.0	0.0
Seaweed Water	0.1	0.3	0.0	0.1	0.0	0.0
Tent Umbrella	0.0	0.1	0.1	0.6	0.0	0.0
Left-Hand	0.0	0.0	0.0	0.0	0.0	0.3
Right hand	0.0	0.0	0.0	0.0	0.0	0.1
Food Drink Com	0.0	0.0	0.0	0.0	0.0	0.0

Table 3. Mean Percent Time for Left-Hand, Right-Hand, and Mouth Surface Contact by Sex (Female = 67, Male = 53).

Variable	Sex	Left-Hand		Right-Hand		Mouth	
		Mean%	Std. Dev	Mean%	Std. Dev	Mean%	Std. Dev
Nothing	F	29.1	13.8	24.3	12.1	96.4	4.7
	M	28.7	12.8	23.1	12.2	95.8	5.6
Plastic Toys	F	18.8	14.7	26.0	16.9	0.1	0.4
	M	20.8	17.5	28.7	18.7	0.0	0.3
Sea Water	F	18.9	15.7	18.4	15.5	0.0	0.1
	M	19.0	15.8	17.5	15.7	0.0	0.1
Sand	F	10.8	11.9	9.3	8.9	0.0	0.1
	M	8.6	9.9	8.8	9.0	0.0	0.1
Sand Seawater	F	4.6	8.3	5.4	9.9	0.0	0.0
	M	5.3	10.9	6.2	10.8	0.0	0.0
Not in view	F	4.4	5.9	3.1	4.6	0.5	1.8
	M	2.4	2.4	2.2	3.1	0.3	0.7
Other Skin	F	4.2	5.9	4.7	6.2	0.0	0.0
	M	5.3	5.9	3.7	4.8	0.0	0.0
Other	F	2.5	3.8	2.8	5.2	0.1	0.7
	M	2.8	3.6	3.8	4.8	0.0	0.1
Own Skin	F	1.4	2.3	1.0	1.6	0.0	0.0
	M	1.0	1.9	1.0	1.9	0.0	0.0
Face	F	1.2	1.6	0.8	1.3	0.0	0.0
	M	1.0	2.0	0.8	1.4	0.0	0.0
Food	F	1.1	3.3	1.1	2.8	1.9	3.5
	M	1.6	3.0	1.8	4.0	2.1	3.6
Clothes	F	1.1	1.5	1.2	1.8	0.0	0.0
	M	1.5	2.3	1.5	2.7	0.0	0.0
Towel Blanket	F	0.7	2.3	0.7	2.8	0.0	0.0
	M	0.3	1.1	0.2	0.6	0.0	0.0
Shells	F	0.5	1.3	0.7	1.9	0.0	0.1
	M	0.4	1.8	0.4	1.9	0.0	0.0
Drinks	F	0.2	0.7	0.0	0.0	0.8	1.6
	M	0.7	2.4	0.0	0.1	1.7	3.1
Mouth	F	0.2	0.6	0.1	0.3	0.0	0.0
	M	0.1	0.4	0.0	0.0	0.0	0.0
Seaweed	F	0.1	0.7	0.3	0.8	0.0	0.0
	M	0.2	0.6	0.3	1.3	0.0	0.0
Seaweed Water	F	0.0	0.2	0.0	0.0	0.0	0.0
	M	0.1	0.4	0.0	0.2	0.0	0.0
Tent Umbrella	F	0.0	0.1	0.1	0.8	0.0	0.0
	M	0.0	0.1	0.1	0.2	0.0	0.0
Metal Toys	F	0.0	0.0	0.0	0.0	0.0	0.0
	M	0.3	2.0	0.2	1.7	0.0	0.0
Left Hand	F	0.0	0.0	0.0	0.0	0.0	0.2
	M	0.0	0.0	0.0	0.0	0.1	0.3
Right Hand	F	0.0	0.0	0.0	0.0	0.0	0.1
	M	0.0	0.0	0.0	0.0	0.0	0.0
Food Drink Com	F	0.0	0.0	0.0	0.0	0.0	0.0
	M	0.0	0.0	0.0	0.0	0.0	0.0

Table 4. Mean Percent Time Various Age Group are Engaged in Surface Touch. (Age Groups: 1 (0-24) = 27, 2 (25-48) = 41, 3 (>48) = 52).

Variable	Age Group	Left-Hand		Right-Hand		Mouth	
		**Mean%**	**Std. Dev**	***Mean%***	***Std. Dev***	**Mean%**	**Std. Dev**
Nothing	1	31.6	16.8	28.5	13.8	94.6	6.0
	2	27.7	12.9	22.9	11.8	96.3	4.6
	3	28.5	11.5	22.1	11.0	96.8	5.0
Plastic Toys	1	20.9	14.7	26.2	14.3	0.2	0.7
	2	21.9	19.3	29.2	19.5	0.0	0.1
	3	17.4	13.5	26.2	18.0	0.0	0.0
Sea Water	1	14.0	12.4	14.6	13.4	0.0	0.1
	2	18.6	16.7	18.2	17.3	0.0	0.2
	3	21.7	15.9	19.6	15.1	0.0	0.0
Other Skin	1	11.4	6.9	8.5	6.9	0.0	0.0
	2	4.2	4.8	3.8	5.0	0.0	0.0
	3	1.7	2.4	2.4	4.0	0.0	0.0
Sand	1	4.9	5.7	5.8	6.5	0.0	0.1
	2	10.6	12.3	10.3	9.7	0.0	0.0
	3	11.8	11.5	9.9	9.1	0.0	0.1
Not in view	1	4.6	5.6	3.3	3.7	0.5	0.9
	2	3.5	4.3	2.8	4.1	0.7	2.2
	3	2.9	4.8	2.3	4.2	0.2	0.8
Other	1	3.7	4.1	4.7	7.3	0.2	1.2
	2	1.9	2.7	2.4	3.3	0.0	0.0
	3	2.6	4.1	3.2	4.6	0.0	0.0
Sand Seawater	1	2.5	5.4	2.8	6.1	0.0	0.0
	2	4.9	11.2	5.4	10.1	0.0	0.0
	3	6.2	9.7	7.5	11.7	0.0	0.0
Food	1	1.8	4.4	1.1	2.7	1.7	3.0
	2	1.4	3.1	1.6	3.8	2.3	3.8
	3	1.1	2.4	1.5	3.4	2.0	3.6
Clothes	1	1.6	2.1	2.0	3.0	0.0.	0.0
	2	1.3	2.5	1.1	1.9	0.0	0.0
	3	1.1	1.1	1.2	2.0	0.0	0.0
Own Skin	1	1.2	2.8	0.9	2.1	0.0	0.0
	2	1.4	2.5	0.6	0.9	0.0	0.0
	3	1.1	1.4	0.8	1.0	0.0	0.0
Face	1	0.6	0.5	0.7	0.9	0.0	0.0
	2	1.2	2.0	0.6	0.9	0.0	0.0
	3	1.3	1.9	1.1	1.7	0.0	0.0
Towel Blanket	1	0.5	1.2	0.6	1.4	0.0	0.0
	2	0.3	1.3	0.2	0.9	0.0	0.0
	3	0.7	2.5	0.7	3.0	0.0	0.0
Mouth	1	0.3	0.8	0.1	0.5	0.0	0.0
	2	0.1	0.1	0.0	0.0	0.0	0.0
	3	0.1	0.4	0.0	0.0	0.0	0.0
Drinks	1	0.2	0.8	0.0	0.0	2.5	4.1
	2	0.2	0.4	0.0	0.0	0.6	0.9
	3	0.7	2.4	0.0	0.1	1.0	1.8
Shells	1	0.1	0.3	0.0	0.0	0.0	0.1
	2	0.8	2.4	0.7	2.4	0.0	0.0
	3	0.4	0.9	0.8	1.9	0.0	0.0

Table S4: continued

Variable	Age Group	Left-Hand		Right-Hand		Mouth	
		**Mean%**	**Std. Dev**	***Mean%***	***Std. Dev***	**Mean%**	**Std. Dev**
Seaweed	1	0.0	0.1	0.2	0.6	0.0	0.0
	2	0.0	0.2	0.1	0.3	0.0	0.0
	3	0.3	1.0	0.5	1.5	0.0	0.0
Metal Toys	1	0.0	0.1	0.0	0.0	0.0	0.0
	2	0.0	0.0	0.0	0.0	0.0	0.0
	3	0.3	2.0	0.2	1.7	0.0	0.0
Seaweed Water	1	0.0	0.0	0.0	0.0	0.0	0.0
	2	0.1	0.3	0.0	0.1	0.0	0.0
	3	0.1	0.4	0.0	0.2	0.0	0.0
Tent Umbrella	1	0.0	0.0	0.0	0.2	0.0	0.0
	2	0.0	0.2	0.2	1.0	0.0	0.0
	3	0.0	0.1	0.0	0.1	0.0	0.0
Mouth	1	0.3	0.8	0.1	0.5	0.0	0.0
	2	0.1	0.1	0.0	0.0	0.0	0.0
	3	0.1	0.4	0.0	0.0	0.0	0.0
Food Drink Com	1	0.0	0.0	0.0	0.0	0.0	0.0
	2	0.0	0.0	0.0	0.0	0.0	0.0
	3	0.0	0.0	0.0	0.0	0.0	0.0
Left-Hand	1	0.0	0.0	0.0	0.0	0.1	0.3
	2	0.0	0.0	0.0	0.0	0.0	0.1
	3	0.0	0.0	0.0	0.0	0.0	0.3
Right-Hand	1	0.0	0.0	0.0	0.0	0.0	0.1
	2	0.0	0.0	0.0	0.0	0.0	0.0
	3	0.0	0.0	0.0	0.0	0.0	0.0

Scheme 5. Mean Percent Time for Left-Hand, Right-Hand and Mouth Surface Contact by Beach. Crandon (Cr) =27, Haulover (Ha) = 33, Seawall (Se) = 31, Stewart (St) = 29.

Variable	Beach	Left-Hand		Right-Hand		Mouth	
		Mean%	Std. Dev	Mean%	Std. Dev	Mean%	Std. Dev
Nothing	Cr.	35.4	12.0	27.4	12.7	94.6	5.1
	Ha.	23.0	9.5	19.8	9.5	94.5	5.3
	Se.	28.2	12.6	26.3	10.4	97.9	4.9
	St.	30.3	16.1	22.2	14.6	97.5	4.4
Plastic Toys	Cr.	20.1	13.7	29.9	18.8	0.1	0.4
	Ha.	11.7	9.7	18.6	12.4	0.0	0.0
	Se.	24.9	20.9	28.1	18.7	0.1	0.4
	St.	22.8	14.6	33.6	17.7	0.1	0.4
Sea Water	Cr.	12.1	10.7	13.7	11.8	0.0	0.2
	Ha.	30.6	17.3	30.9	18.4	0.0	0.1
	Se.	18.4	13.1	14.7	10.3	0.0	0.0
	St.	12.5	12.7	10.8	10.9	0.0	0.0
Other Skin	Cr.	4.8	6.1	3.4	4.5	0.0	0.0
	Ha.	4.5	6.8	3.1	4.2	0.0	0.0
	Se.	4.9	5.3	5.7	7.0	0.0	0.0
	St.	4.7	5.6	4.7	6.1	0.0	0.0
Sand	Cr.	11.0	11.3	10.4	9.2	0.0	0.2
	Ha.	10.5	12.9	9.8	9.9	0.0	0.1
	Se.	7.2	8.2	7.7	7.7	0.0	0.1
	St.	10.8	11.4	8.7	8.9	0.0	0.0
Not in view	Cr.	3.9	3.7	3.5	3.1	0.9	1.9
	Ha.	7.2	6.9	5.9	5.7	0.8	2.0
	Se.	1.1	1.5	0.4	0.6	0.0	0.0
	St.	1.6	1.4	0.6	0.9	0.0	0.0
Other	Cr.	3.4	4.0	5.6	8.3	0.0	0
	Ha.	3.9	4.6	4.5	3.9	0.2	1.0
	Se.	1.5	2.7	1.6	2.5	0.0	0.0
	St.	1.5	2.5	1.5	2.2	0.0	0.0
Sand Seawater	Cr.	0.5	0.8	0.7	1.4	0.0	0.0
	Ha.	0.8	1.1	0.7	1.3	0.0	0.0
	Se.	8.6	10.5	11.1	13.4	0.0	0.0
	St.	9.8	13.6	10.4	11.9	0.0	0.0
Food	Cr.	2.3	4.5	1.8	3.6	2.2	2.8
	Ha.	2.0	3.5	2.7	4.4	3.5	4.4
	Se.	0.4	1.1	0.1	0.4	0.8	1.9
	St.	0.7	2.4	0.9	3.1	1.5	3.9
Clothes	Cr.	1.7	2.6	1.7	3.0	0.0	0.0
	Ha.	0.8	1.3	0.7	1.4	0.0	0.0
	Se.	1.5	2.2	1.1	1.5	0.0	0.0
	St.	1.2	1.2	2.1	2.8	0.0	0.0
Own Skin	Cr.	1.1	2.4	0.3	0.4	0.0	0.0
	Ha.	1.3	2.3	0.6	1.0	0.0	0.0
	Se.	0.9	0.8	0.6	0.7	0.0	0.0
	St.	1.7	2.8	1.6	2.1	0.0	0.0
Face	Cr.	1.0	1.5	0.6	1.1	0.0	0.0
	Ha.	1.0	2.0	0.5	0.7	0.0	0.0
	Se.	1.4	2.2	1.4	2.0	0.0	0.0
	St.	1.0	1.1	0.9	1.1	0.0	0.0

Table 5. continued.

Variable	Beach	Left-Hand		Right-Hand		Mouth	
		Mean%	Std. Dev	Mean%	Std. Dev	Mean%	Std. Dev
Towel Blanket	Cr.	0.3	0.8	0.3	0.8	0.0	0.0
	Ha.	1.3	3.2	0.5	1.2	0.0	0.0
	Se.	0.2	0.8	0.3	1.2	0.0	0.0
	St.	0.3	1.1	0.8	3.9	0.0	0.0
Mouth	Cr.	0.2	0.6	0.0	0.0	0.0	0.0
	Ha.	0.1	0.4	0.0	0.1	0.0	0.0
	Se.	0.1	0.7	0.0	0.0	0.0	0.0
	St.	0.0	0.1	0.1	0.4	0.0	0.0
Drinks	Cr.	1.3	3.2	0.0	0.2	2.0	2.4
	Ha.	0.1	0.2	0.0	0.0	0.9	1.5
	Se.	0.0	0.1	0.0	0.0	1.2	3.7
	St.	0.5	1.1	0.0	0.0	0.9	1.3
Shells	Cr.	0.1	0.3	0.1	0.3	0.0	0.0
	Ha.	1.1	2.5	1.3	3.0	0.0	0.1
	Se.	0.6	1.4	0.8	1.6	0.0	0.0
	St.	0.0	0.0	0.0	0.2	0.0	0.0
Seaweed	Cr.	0.0	0.0	0.1	0.3	0.0	0.0
	Ha.	0.1	0.2	0.0	0.2	0.0	0.0
	Se.	0.0	0.2	0.0	0.2	0.0	0.0
	St.	0.5	1.3	0.0	0.0	0.0	0.0
Metal Toys	Cr.	0.6	2.8	0.5	2.4	0.0	0.0
	Ha.	0.0	0.1	0.0	0.1	0.0	0.0
	Se.	0.0	0.0	0.0	0.0	0.0	0.0
	St.	0.0	0.0	0.0	0.0	0.0	0.0
Seaweed Water	Cr.	0.1	0.4	0.0	0.2	0.0	0.0
	Ha.	0.0	0.0	0.0	0.1	0.0	0.0
	Se.	0.0	0.0	0.0	0.0	0.0	0.0
	St.	0.1	0.5	0.0	0.0	0.0	0.0
Tent Umbrella	Cr.	0.0	0.1	0.1	0.2	0.0	0.0
	Ha.	0.0	0.1	0.2	1.1	0.0	0.0
	Se.	0.0	0.0	0.0	0.2	0.0	0.0
	St.	0.0	0.2	0.0	0.1	0.0	0.0
Left-Hand	Cr.	0.0	0.0	0.0	0.0	0.2	0.5
	Ha.	0.0	0.0	0.0	0.0	0.0	0.2
	Se.	0.0	0.0	0.0	0.0	0.0	0.0
	St.	0.0	0.0	0.0	0.0	0.0	0.0
Right-Hand	Cr.	0.0	0.0	0.0	0.0	0.0	0.0
	Ha.	0.0	0.0	0.0	0.0	0.0	0.1
	Se.	0.0	0.0	0.0	0.0	0.0	0.0
	St.	0.0	0.0	0.0	0.0	0.0	0.0
Food Drink Cont.	Cr.	0.0	0.0	0.0	0.0	0.0	0.0
	Ha.	0.0	0.0	0.0	0.0	0.0	0.0
	Se.	0.0	0.0	0.0	0.0	0.0	0.0
	St.	0.0	0.0	0.0	0.0	0.0	0.0

Scheme 6. Surface Contacts for Left-Hand, Right-Hand and Mouth while Engaged in Activities

Activity	Surface	Left-Hand		Right-Hand		Mouth	
		Mean%	Std. Dev	Mean%	Std. Dev	Mean%	Std. Dev
Digging	Metal Toys	0.1	0.6	0.0	0.0	0.0	0.0
	Sand Seawater	2.2	6.3	0.1	0.3	0.0	0.0
	Plastic Toys	6.0	6.2	2.9	3.1	0.0	0.1
	Sand	6.8	9.0	0.1	0.3	0.0	0.0
	Towel Blanket	0.1	0.6	0.2	1.7	0.0	0.0
	Sea Water	0.5	2.1	0.1	0.3	0.0	0.0
	Not in view	0.8	1.5	0.5	1.1	0.1	0.8
	Shells	0.2	0.8	0.2	0.8	0.0	0.0
	Nothing	5.2	5.7	0.1	0.3	22.5	16.2
	Other	0.2	0.6	0.2	1.0	0.0	0.0
	Seaweed	0.0	0.2	0.1	0.3	0.0	0.0
	Seaweed Water	0.0	0.0	0.1	0.3	0.0	0.0
	Own Skin	0.3	1.2	0.2	0.6	0.0	0.0
	Other Skin	0.1	0.3	0.1	0.5	0.0	0.0
	Drinks	0.0	0.1	0.0	0.0	0.1	0.5
	Mouth	0.0	0.0	0.0	0.0	0.0	0.0
	Food	0.0	0.0	0.0	0.1	0.1	0.5
	Clothes	0.1	0.1	0.1	0.3	0.0	0.0
	Face	0.1	0.3	0.1	0.3	0.0	0.0
	Tent Umbrella	0.0	0.0	0.0	0.0	0.0	0.0
Not in view	Sea Water	0.0	0.2	0.0	0.4	0.0	0.0
	Plastic Toys	0.1	0.6	0.2	1.0	0.0	0.0
	Not in view	0.1	0.4	0.1	0.4	0.0	0.2
	Nothing	0.1	0.3	0.1	0.2	0.3	1.0
	Sand	0.0	0.0	0.0	0.1	0.0	0.0
	Other	0.0	0.0	0.0	0.0	0.0	0.0
	Seaweed	0.0	0.0	0.0	0.1	0.0	0.0
	Seaweed Water	0.1	0.7	0.0	0.1	0.0	0.0
	Food	0.0	0.0	0.0	0.0	0.0	0.0
	Other Skin	[illegible]	0.0	[illegible]	1.8	0.0	0.0
	Drinks	0.0	0.0	0.0	0.0	0.0	0.0
	Sand Seawater	0.0	0.0	0.0	0.0	0.0	0.0
	Face	0.0	0.0	0.0	0.0	0.0	0.0
	Clothes	0.0	0.0	0.3	1.8	0.0	0.0
	Towel Blanket	0.0	0.0	0.0	0.0	0.0	0.0
Running	Other Skin	0.1	0.3	0.1	0.4	0.0	0.0
	Plastic Toys	0.8	2.5	1.1	1.7	0.0	0.0
	Sand Seawater	0.1	0.6	0.1	0.6	0.0	0.0
	Nothing	1.2	1.6	0.9	1.5	0.0	0.0
	Sea Water	0.2	0.7	0.2	0.7	0.0	0.0
	Shells	0.0	0.1	0.0	0.1	0.0	0.0
	Clothes	0.0	0.2	0.1	0.3	0.0	0.0
	Not in view	0.0	0.2	0.0	0.0	2.6	3.4
	Sand	0.1	0.2	0.2	0.9	0.0	0.0
	Towel Blanket	0.0	0.0	0.0	0.0	0.0	0.0
	Other	0.0	0.1	0.0	0.1	0.0	0.0

Table 6. continued.

Activity	Surface	Left-Hand		Right-Hand		Mouth	
		Mean%	Std. Dev	Mean%	Std. Dev	Mean%	Std. Dev
	Seaweed	0.0	0.0	0.0	0.0	0.0	0.0
	Seaweed Water	0.0	0.0	0.1	0.6	0.0	0.0
	Tent Umbrella	0.0	0.0	0.0	0.1	0.0	0.0
	Drinks	0.0	0.0	0.0	0.0	0.0	0.0
	Food	0.0	0.1	0.0	0.0	0.0	0.1
	Face	0.0	0.0	0.0	0.1	0.0	0.0
	Other Skin	0.0	0.0	0.1	0.4	0.0	0.0
	Own Skin	0.0	0.0	0.0	0.1	0.0	0.0
	Metal Toys	0.0	0.0	0.0	0.1	0.0	0.0
Sitting	Other Skin	0.7	2.1	0.4	1.5	0.0	0.0
	Plastic Toys	2.6	5.0	3.4	6.4	0.0	0.1
	Towel Blanket	0.4	1.3	0.0	0.0	0.0	0.0
	Sea Water	0.3	1.6	0.4	1.8	0.0	0.0
	Sand Seawater	0.1	0.6	0.4	1.3	0.0	0.0
	Not in view	1.2	3.4	1.1	3.2	0.2	0.6
	Sand	1.1	2.2	0.7	1.5	0.0	0.0
	Other	0.9	2.2	1.3	4.2	0.0	0.3
	Clothes	0.2	0.8	0.3	1.2	0.0	0.0
	Nothing	2.6	3.5	2.2	2.9	10.0	12.3
	Food	0.8	2.6	0.7	2.4	1.0	2.6
	Own Skin	0.3	0.9	0.2	0.5	0.0	0.0
	Shells	0.0	0.1	0.3	1.8	0.0	0.0
	Drinks	0.2	1.5	0.0	0.0	0.5	1.4
	Seaweed	0.0	0.1	3.4	6.4	0.0	0.0
	Seaweed Water	0.0	0.0	0.7	1.5	0.0	0.0
	Tent Umbrella	0.0	0.0	0.0	0.1	0.0	0.0
	Face	0.1	0.3	0.1	0.4	0.0	0.0
	Mouth	0.0	0.2	0.0	0.2	0.0	0.0
	Left-Hand	0.0	0.0	0.0	0.0	0.0	0.0
	Right-Hand	0.0	0.0	0.0	0.0	0.0	0.0
	Food Drink Cont.	0.0	0.0	0.0	0.0	0.0	0.0
	Metal Toys	0.0	0.0	0.0	0.1	0.0	0.0
Sleeping	Not in view	0.0	0.0	0.0	0.1	0.0	0.0
	Towel Blanket	0.0	0.1	0.0	0.0	0.0	0.0
	Other Skin	0.0	0.0	0.0	0.0	0.0	0.0
	Other	0.0	0.4	0.0	0.2	0.0	0.0
	Own Skin	0.0	0.1	0.0	0.0	0.0	0.0
	Clothes	0.0	0.0	0.0	0.0	0.0	0.0
	Face	0.0	0.1	0.0	0.0	0.0	0.0
	Food	0.0	0.0	0.0	0.5	0.1	0.6
	Drinks	0.0	0.0	0.2	0.6	0.0	0.0
	Nothing	0.0	0.3	0.0	0.1	0.1	0.4
	Plastic Toys	0.0	0.0	0.0	0.2	0.0	0.0
	Sand	0.0	0.0	0.0	0.0	0.0	0.0
Standing	Seaweed	0.0	0.1	0.0	0.1	0.0	0.0
	Seaweed Water	0.0	0.0	0.0	0.0	0.0	0.0
	Plastic Toys	2.6	3.9	3.6	5.6	0.0	0.0
	Shells	0.1	0.7	0.0	0.0	0.0	0.0
	Sea Water	0.3	1.0	0.3	1.0	0.0	0.0
	Nothing	5.8	6.3	4.8	5.4	11.3	0.0
	Other Skin	0.3	1.2	0.3	0.9	0.0	0.0
	Other	0.9	1.6	1.0	1.8	0.0	0.0

	Sand Seawater	0.1	0.3	0.1	0.6	0.0	0.0

Table 6. continued.

Activity	Surface	Left-Hand		Right-Hand		Mouth	
		Mean%	Std. Dev	Mean%	Std. Dev	Mean%	Std. Dev
	Food	0.3	1.0	0.4	1.5	0.6	0.0
	Sand	0.5	1.0	0.6	1.0	0.0	0.0
	Not in view	0.4	0.9	0.4	0.9	0.0	0.0
	Tent Umbrella	0.0	0.1	0.0	0.0	0.0	0.0
	Drinks	0.1	0.3	0.0	0.1	0.3	0.0
	Towel Blanket	0.0	0.2	0.0	0.0	0.0	0.0
	Clothes	0.2	0.5	0.3	0.7	0.0	0.0
	Own Skin	0.2	0.5	0.1	0.3	0.0	0.0
	Face	0.1	0.5	0.0	0.1	0.0	0.0
	Metal Toys	0.0	0.1	0.0	0.1	0.0	0.0
	Mouth	0.0	0.0	0.0	0.0	0.0	0.0
	Left-Hand	0.0	0.0	0.0	0.0	0.0	0.0
	Right-Hand	0.0	0.0	0.0	0.0	0.0	0.0
	Food Drink Com	0.0	0.0	0.0	0.0	0.0	0.0
Swimming	Nothing	0.0	0.0	0.0	0.0	0.0	0.0
	Seawater	0.0	0.0	0.0	0.0	0.0	0.0
	Sand Seawater	0.0	0.0	0.0	0.0	0.0	0.0
	Other Skin	0.0	0.0	0.0	0.0	0.0	0.0
	Clothes	0.0	0.0	0.0	0.0	0.0	0.0
	Plastic Toys	0.0	0.0	0.0	0.0	0.0	0.0
Wading	Sea Water	17.0	14.7	16.3	14.5	0.0	0.0
	Other Skin	3.0	4.0	3.0	4.5	0.0	0.0
	Plastic Toys	5.9	9.3	8.1	10.5	0.0	0.0
	Sand Seawater	2.0	4.1	2.7	5.8	0.0	0.0
	Nothing	9.6	8.4	8.7	7.8	41.0	0.0
	Sand	0.7	1.5	0.7	1.6	0.0	0.0
	Not in view	0.7	1.3	0.4	0.8	0.0	0.0
	Seaweed Water	0.1	0.3	0.0	0.0	0.0	0.0
	Other	0.3	0.8	0.3	0.9	0.0	0.0
	Food	0.1	0.8	0.1	0.6	0.2	0.0
	Towel Blanket	0.0	0.0	0.0	0.0	0.0	0.0
	Drinks	0.0	0.2	0.0	0.0	0.2	0.0
	Clothes	0.6	1.2	0.5	1.4	0.0	0.0
	Metal Toys	0.0	0.0	0.0	0.0	0.0	0.0
	Seaweed	0.1	0.4	0.0	0.0	0.0	0.0
	Shells	0.1	0.3	0.0	0.0	0.0	0.0
	Mouth	0.1	0.4	0.5	1.4	0.0	0.0
	Own Skin	0.3	0.6	0.2	0.4	0.0	0.0
	Face	0.7	1.4	0.5	1.1	0.0	0.0
	Left-Hand	0.0	0.0	0.0	0.0	0.0	0.0
	Right-Hand	0.0	0.0	0.0	0.0	0.0	0.0
Walking	Other Skin	0.5	1.6	0.2	0.5	0.0	0.0
	Sand Seawater	0.3	1.0	1.8	3.0	0.0	0.0
	Sea Water	0.4	1.5	0.6	1.3	0.0	0.0
	Plastic Toys	1.7	2.2	0.5	1.6	0.0	0.0
	Nothing	4.3	4.6	4.3	5.5	0.0	0.0
	Seaweed	0.0	0.3	0.2	0.4	0.0	0.0
	Shells	0.1	0.2	0.8	1.7	0.0	0.0
	Other	0.3	1.1	0.4	1.5	0.0	0.0
	Metal Toys	0.1	0.6	0.1	0.9	0.0	0.0

	Sand	0.5	1.7	0.6	1.2	0.0	0.0
	Food	0.1	0.5	0.1	1.2	0.1	0.0
	Drinks	0.0	0.2	0.0	0.0	0.0	0.0

Table 6. continued.

Activity	Surface	Left-Hand		Right-Hand		Mouth	
		Mean%	Std. Dev	Mean%	Std. Dev	Mean%	Std. Dev
	Not in view	0.1	0.3	0.2	0.4	0.0	0.0
	Clothes	0.1	0.3	0.1	0.3	0.0	0.0
	Towel Blanket	0.0	0.1	0.2	0.8	0.0	0.0
	Own Skin	0.1	0.2	0.1	0.2	0.0	0.0
	Face	0.1	0.2	0.1	0.2	0.0	0.0
	Seaweed Water	0.0	0.0	0.0	0.1	0.0	0.0
	Tent Umbrella	0.0	0.0	16.9	15.9	0.0	0.0
	Mouth	0.0	0.0	0.0	0.0	0.0	0.0
	Left-Hand	0.0	0.0	0.0	0.0	0.0	0.0
	Right-Hand	0.0	0.0	0.0	0.0	0.0	0.0
	Food Drink Cont.	0.0	0.0	0.0	0.0	0.0	0.0

Scheme 7. Surfaces Contacts by Left-Hand, Right-Hand and Mouth While In Beach Locations.

Location	Surface	Left-Hand		Right-Hand		Mouth	
		Mean%	Std. Dev	Mean%	Std. Dev	Mean	Std Dev
Back Beach	Not in view	0.0	0.0	0.0	0.0	0.0	0.0
	Other Skin	0.0	0.1	0.0	0.0	0.0	0.0
	Plastic Toys	0.0	0.3	0.0	0.1	0.0	0.0
	Own Skin	0.0	0.0	0.0	0.0	0.0	0.0
	Face	0.0	0.1	0.0	0.0	0.0	0.0
	Nothing	0.1	0.6	0.1	0.7	0.2	1.0
	Metal Toys	0.0	0.2	0.0	0.3	0.0	0.0
	Sand	0.0	0.2	0.0	0.0	0.0	0.0
	Shells	0.0	0.0	0.0	0.0	0.0	0.0
	Other	0.0	0.2	0.0	0.0	0.0	0.0
	Clothes	0.0	0.0	0.0	0.0	0.0	0.0
	Tent Umbrella	0.0	0.0	0.0	0.0	0.0	0.0
Back Beach Trough	Food	0.0	0.3	0.0	0.0	0.1	1.0
	Clothes	0.0	0.1	0.0	0.1	0.0	0.0
	Drinks	0.0	0.0	0.0	0.0	0.0	0.2
	Face	0.0	0.0	0.0	0.2	0.0	0.0
	Not in view	0.0	0.1	0.0	0.1	0.0	0.0
	Other	0.1	0.8	0.0	0.0	0.0	0.0
	Other Skin	0.0	0.0	0.2	1.7	0.0	0.0
	Own Skin	0.0	0.0	0.0	0.1	0.0	0.0
	Sand Seawater	0.0	0.0	0.1	1.2	0.0	0.0
	Plastic Toys	0.1	1.6	0.2	2.6	0.0	0.0
	Nothing	0.1	1.0	0.1	1.5	0.6	6.7
	Sand	0.3	2.9	0.0	0.0	0.0	0.0
	Seaweed	0.0	0.0	0.0	0.1	0.0	0.0
	Seawater	0.0	0.0	0.1	1.5	0.0	0.0
Berm Crest	Food	0.5	1.8	0.0	0.0	0.8	2.4
	Tent Umbrella	0.0	0.1	0.0	0.0	0.0	0.0
	Towel Blanket	0.2	1.2	0.0	0.0	0.0	0.0
	Other	0.8	2.4	0.0	0.0	0.0	0.0
	Sand Seawater	0.1	0.5	0.1	1.2	0.0	0.0
	Mouth	0.0	0.1	0.0	0.0	0.0	0.0
	Own Skin	0.4	1.6	0.0	0.1	0.0	0.0
	Seaweed	0.0	0.1	0.0	0.1	0.0	0.0
	Not in view	0.6	2.3	0.0	0.1	0.1	0.4
	Shells	0.1	0.6	0.0	0.0	0.0	0.0
	Seawater	0.2	1.6	0.1	1.5	0.0	0.0
	Seaweed Water	0.0	0.0	0.0	0.0	0.0	0.0
	Other Skin	0.4	1.6	0.2	1.7	0.0	0.0
	Nothing	4.7	7.8	0.1	1.5	12.0	17.8
	Face	0.1	0.3	0.0	0.2	0.0	0.0
	Metal Toys	0.1	0.8	0.0	0.0	0.0	0.0
	Sand	1.5	4.4	0.0	0.0	0.0	0.0
	Plastic Toys	2.7	6.3	0.2	2.6	0.0	0.2
	Clothes	0.2	0.8	0.0	0.1	0.0	0.0
	Drinks	0.1	0.3	0.0	0.0	0.4	1.2
	Left-Hand	0.0	0.0	0.0	0.0	0.0	0.0
	Right-Hand	0.0	0.0	0.0	0.0	0.0	0.0
	Food Drink Cont.	0.0	0.0	0.0	0.0	0.0	0.0

Table 7. continued.

Location	Surface	Left-Hand Mean%	Left-Hand Std. Dev	Right-Hand Mean%	Right-Hand Std. Dev	Mouth Mean%	Mouth Std. Dev
Boardwalk	Clothes	0.0	0.0	0.0	0.0	0.0	0.0
	Drinks	0.0	0.0	0.0	0.0	0.0	0.0
	Not in view	0.0	0.1	0.0	0.0	0.0	0.0
	Nothing	0.1	0.7	0.0	0.0	0.2	1.9
	Other	0.0	0.1	0.0	0.2	0.0	0.0
	Own Skin	0.0	0.0	0.0	0.0	0.0	0.0
	Plastic Toys	0.1	1.4	0.0	0.0	0.0	0.0
	Sand	0.0	0.5	0.2	2.5	0.0	0.0
	Seawater	0.0	0.1	0.0	0.0	0.0	0.0
	Shells	0.0	0.0	0.0	0.1	0.0	0.0
Dune Areas	Plastic Toys	0.0	0.1	0.0	0.0	0.0	0.0
	Tent Umbrella	0.0	0.0	0.0	0.0	0.0	0.0
	Sand	0.0	0.0	0.0	0.0	0.0	0.0
	Other	0.0	0.0	0.0	0.1	0.0	0.0
	Nothing	0.0	0.3	0.0	0.2	0.0	0.4
	Food	0.0	0.3	0.0	0.1	0.0	0.0
	Not in view	0.0	0.1	0.0	0.2	0.0	0.0
	Clothes	0.0	0.0	0.0	0.0	0.0	0.0
	Mouth	0.0	0.0	0.0	0.0	0.0	0.0
	Own Skin	0.0	0.0	0.0	0.0	0.0	0.0
	Drinks	0.0	0.0	0.0	0.0	0.0	0.0
Dune Ridge	Mouth	0.0	0.0	0.0	0.0	0.0	0.0
	Shells	0.0	0.0	0.0	0.0	0.0	0.0
	Drinks	0.0	0.0	0.0	0.0	0.5	1.6
	Tent Umbrella	0.0	0.0	0.0	0.0	0.0	0.0
	Seawater	0.0	0.0	0.0	0.0	0.0	0.1
	Sand	0.0	0.0	0.0	0.0	0.0	0.0
	Own Skin	0.0	0.0	0.0	0.0	0.0	0.0
	Other Skin	0.0	0.0	0.0	0.0	0.0	0.0
	Not in view	0.0	0.1	0.0	0.2	0.2	0.9
	Face	0.0	0.0	0.0	0.0	0.0	0.0
	Nothing	0.0	0.3	0.0	0.2	16.0	20.6
	Food	0.0	0.3	0.0	0.1	0.6	1.7
	Other	0.0	0.0	0.0	0.1	0.0	0.3
	Plastic Toys	0.0	0.1	0.0	0.0	0.0	0.0
	Towel Blanket	0.0	0.0	0.0	0.0	0.0	0.0
	Clothes	0.0	0.0	0.0	0.0	0.0	0.0
	Metal Toys	0.0	0.0	0.0	0.0	0.0	0.0
	Sand Seawater	0.0	0.0	0.0	0.0	0.0	0.0
	Seaweed	0.0	0.0	0.0	0.0	0.0	0.0
	Left-Hand	0.0	0.0	0.0	0.0	0.0	0.2
	Right-Hand	0.0	0.0	0.0	0.0	0.0	0.0
	Food Drink Cont.	0.0	0.0	0.0	0.0	0.0	0.0
Intertidal	Seaweed	0.1	0.5	0.1	0.5	0.0	0.0
	Seaweed Water	0.0	0.0	1.0	2.3	0.0	0.0
	Other	0.2	0.8	0.2	0.7	0.0	0.0
	Food	0.1	0.4	0.1	0.9	0.4	1.6
	Sand	3.2	5.8	3.2	5.2	0.0	0.0
	Clothes	0.1	0.4	0.1	0.6	0.0	0.0
	Sand Seawater	1.3	3.6	1.1	3.1	0.0	0.0
	Own Skin	0.2	0.8	0.2	0.8	0.0	0.0
	Mouth	0.0	0.1	0.0	0.0	0.0	0.0

	Face	0.1	0.3	0.1	0.3	0.0	0.0
	Plastic Toys	4.7	7.2	6.3	9.9	0.0	0.0

Table 7. continued.

Location	Surface	Left-Hand		Right-Hand		Mouth	
		**Mean%**	**Std. Dev**	**Mean%**	**Std. Dev**	**Mean%**	**Std. Dev**
	Nothing	5.6	7.5	4.5	5.7	19.3	20.0
	Other Skin	0.6	1.8	0.4	1.7	0.0	0.0
	Not in view	0.4	0.9	0.3	1.0	0.1	0.4
	Seawater	0.9	2.0	1.0	2.3	0.0	0.0
	Metal Toys	0.0	0.0	0.0	0.1	0.0	0.0
	Shells	0.1	0.5	0.1	0.3	0.0	0.0
	Drinks	0.0	0.1	0.0	0.0	0.1	0.6
	Towel Blanket	0.0	0.5	0.0	0.0	0.0	0.0
Not in view	Clothes	0.0	0.0	0.0	0.0	0.0	0.0
	Face	0.0	0.0	0.0	0.0	0.0	0.0
	Drinks	0.0	0.0	0.0	0.0	0.0	0.0
	Other	0.0	0.0	0.0	0.0	0.0	0.0
	Seawater	0.0	0.1	0.0	0.0	0.0	0.0
	Plastic Toys	0.0	0.3	0.0	0.0	0.0	0.0
	Not in view	0.1	0.5	0.0	0.0	0.0	0.0
	Sand	0.0	0.1	0.0	0.0	0.0	0.0
	Food	0.0	0.0	0.0	0.0	0.0	0.0
	Sand Seawater	0.0	0.1	0.0	0.0	0.0	0.0
	Nothing	0.0	0.3	0.0	0.0	0.2	0.0
	Towel Blanket	0.0	0.0	0.0	0.0	0.0	0.0
Rock Jetty	Not in view	0.0	0.0	0.0	0.0	0.0	0.0
	Own Skin	0.0	0.1	0.0	0.0	0.0	0.0
	Other Skin	0.0	0.4	0.0	0.1	0.0	0.0
	Drinks	0.0	0.0	0.0	0.0	0.0	0.0
	Face	0.0	0.1	0.0	0.0	0.0	0.0
	Nothing	0.0	0.2	0.1	0.5	0.1	0.0
	Seawater	0.0	0.0	0.0	0.4	0.0	0.0
Rock Wall	Not in view	0.0	0.0	0.0	0.1	0.0	0.0
	Other	0.0	0.2	0.0	0.3	0.0	0.0
	Sand	0.0	0.0	0.0	0.0	0.0	0.0
	Plastic Toys	0.0	0.1	0.0	0.0	0.0	0.0
Sand Bars	Clothes	0.0	0.0	0.0	0.1	0.0	0.0
	Food	0.0	0.0	0.0	0.0	0.0	0.0
	Own Skin	0.0	0.0	0.0	0.0	0.0	0.0
	Face	0.0	0.2	0.0	0.1	0.0	0.0
	Seawater	0.0	0.0	0.1	0.4	0.0	0.0
	Sand Seawater	0.0	0.2	0.0	0.0	0.0	0.0
	Plastic Toys	0.3	1.7	0.5	3.0	0.0	0.0
	Nothing	0.5	2.6	0.3	1.8	1.3	0.0
	Sand	0.3	2.3	0.3	1.8	0.0	0.0
	Not in view	0.0	0.2	0.0	0.2	0.0	0.0
	Other Skin	0.0	0.1	0.0	0.1	0.0	0.0

Table 7. continued.

Location	Surface	Left-Hand		Right-Hand		Mouth	
		Mean%	Std. Dev	Mean%	Std. Dev	Mean%	Std. Dev
Seawater	Towel Blanket	0.0	0.0	0.1	1.4	0.0	0.0
	Food	0.2	1.0	0.1	0.8	0.0	0.0
	Metal Toys	0.0	0.0	0.0	0.0	0.0	0.0
	Other	0.4	1.0	0.3	0.8	0.0	0.0
	Face	0.8	1.3	0.6	1.1	0.0	0.0
	Clothes	0.7	1.6	0.7	1.6	0.0	0.0
	Seawater	17.5	13.6	16.2	14.1	0.0	0.0
	Sand	1.3	2.9	1.2	2.5	0.0	0.0
	Plastic Toys	7.1	10.0	9.6	10.9	0.0	0.0
	Sand Seawater	3.6	7.3	4.3	8.8	0.0	0.0
	Own Skin	0.3	0.6	0.2	0.4	0.0	0.0
	Nothing	11.6	9.8	9.9	8.6	0.0	0.0
	Other Skin	3.2	3.8	3.1	4.5	0.0	0.0
	Not in view	1.0	1.8	0.7	1.2	0.0	0.0
	Drinks	0.0	0.2	0.0	0.0	0.3	0.0
	Shells	0.1	0.3	0.1	0.6	0.0	0.0
	Mouth	0.1	0.4	0.0	0.2	0.0	0.0
	Seaweed	0.1	0.4	0.2	0.8	0.0	0.0
	Seaweed Water	0.1	0.3	0.0	0.1	0.0	0.0
	Right Hand	0.0	0.0	0.0	0.0	0.0	0.0
	Left Hand	0.0	0.0	0.0	0.0	0.0	0.0
